# Phototropin2 3’UTR overlaps with the *AT5G58150* gene encoding an inactive RLK kinase

**DOI:** 10.1186/s12870-024-04732-2

**Published:** 2024-01-18

**Authors:** Justyna Łabuz, Agnieszka Katarzyna Banaś, Piotr Zgłobicki, Aneta Bażant, Olga Sztatelman, Aleksandra Giza, Hanna Lasok, Aneta Prochwicz, Anna Kozłowska-Mroczek, Urszula Jankowska, Paweł Hermanowicz

**Affiliations:** 1 Malopolska Centre of Biotechnology, Jagiellonian University, Gronostajowa 7A, 30-387 Kraków, Poland; 2 Department of Plant Biotechnology, Faculty of Biochemistry, Biophysics and Biotechnology, Jagiellonian University, Gronostajowa 7, 30-387 Kraków, Poland; 3 Institute of Biochemistry and Biophysics, Polish Academy of Sciences, Pawińskiego 5a, 02-106 Warsaw, Poland; 4 Doctoral School of Exact and Natural Sciences, Jagiellonian University, Łojasiewicza 11, 30-348 Kraków, Poland

**Keywords:** *Arabidopsis*, AT5G58150, LRR RLK, Kinase, phototropin2, Salt stress, Osmotic stress

## Abstract

**Background:**

This study examines the biological implications of an overlap between two sequences in the *Arabidopsis* genome, the 3’UTR of the *PHOT2* gene and a putative *AT5G58150* gene, encoded on the complementary strand. AT5G58150 is a probably inactive protein kinase that belongs to the transmembrane, leucine-rich repeat receptor-like kinase family. Phot2 is a membrane-bound UV/blue light photoreceptor kinase. Thus, both proteins share their cellular localization, on top of the proximity of their *loci*.

**Results:**

The extent of the overlap between 3’UTR regions of *AT5G58150* and *PHOT2* was found to be 66 bp, using RACE PCR. Both the *at5g58150* T-DNA SALK_093781C (with insertion in the promoter region) and 35S::AT5G58150-GFP lines overexpress the *AT5G58150* gene. A detailed analysis did not reveal any substantial impact of *PHOT2* or *AT5G58150* on their mutual expression levels in different light and osmotic stress conditions. AT5G58150 is a plasma membrane protein, with no apparent kinase activity, as tested on several potential substrates. It appears not to form homodimers and it does not interact with PHOT2. Lines that overexpress *AT5G58150* exhibit a greater reduction in lateral root density due to salt and osmotic stress than wild-type plants, which suggests that AT5G58150 may participate in root elongation and formation of lateral roots. In line with this, mass spectrometry analysis identified proteins with ATPase activity, which are involved in proton transport and cell elongation, as putative interactors of AT5G58150. Membrane kinases, including other members of the LRR RLK family and BSK kinases (positive regulators of brassinosteroid signalling), can also act as partners for AT5G58150.

**Conclusions:**

AT5G58150 is a membrane protein that does not exhibit measurable kinase activity, but is involved in signalling through interactions with other proteins. Based on the interactome and root architecture analysis, AT5G58150 may be involved in plant response to salt and osmotic stress and the formation of roots in *Arabidopsis.*

**Supplementary Information:**

The online version contains supplementary material available at 10.1186/s12870-024-04732-2.

## Background

Phototropin2 (phot2) is an ultraviolet and blue light-sensitive plant photoreceptor [1]. It consists of photosensory domains called light oxygen and voltage-activated (LOV) at the N – terminus. LOV domains bind flavin mononucleotides as chromophores and regulate protein interactions, as they belong to the PAS (Per, ARNT, Sim) family [2]. The C – terminal part contains a light-activated serine-threonine kinase [3]. Phototropin2 is plasma membrane-bound in darkness, even though it lacks a transmembrane domain [4]. Upon blue light irradiation, a fraction of the photoreceptor moves to the Golgi apparatus [4]. In *Arabidopsis thaliana,* two phototropins phot1 and phot2, redundantly regulate phototropism [5], stomata opening [6], leaf blade formation [7], leaf positioning [8], and chloroplast accumulation [5]. Nuclear movements [9, 10], chloroplast avoidance [5], and dark positioning are controlled only by phot2 [11].

According to data deposited in TAIR, the Arabidopsis Information Resource (www.arabidopsis.org), the 3’UTR of the *Arabidopsis PHOT2* gene is very long, over 3000 bp. In the genomic region corresponding to this 3’UTR, a putative gene, *At5g58150*, is encoded on the complementary strand. Long 3’UTR of *PHOT2* genes seem to be characteristic of *Arabidopsis thaliana* and other members of the *Brassicaceae* family (*A. lyrata, A. halleri,* Turnip mustard, Drummond’s rockcress, Napa cabbage)*,* as rice phototropin2 has a short 3’UTR with no overlapping gene sequences, encoded on the complementary strand (analysis with VISTA-Point, [12], https://pipeline.lbl.gov/) (Fig. S1). Such arrangements in the genome of mustard family plants suggest that *AT5G58150* may form a cis-natural sense-antisense transcript (cis-NAT) pair with *AT5G58140* (*PHOT2*)*.* This may result in natural antisense transcript small interfering RNA (nat-siRNAs) generation between transcripts, leading to the degradation or alternative mRNA splicing. In *Arabidopsis* protein-coding cis-NAT pairs are highly and broadly expressed, as the production of corresponding nat-siRNAs is limited [13]. The genome proximity of two genes encoded on opposite DNA strands may facilitate or enhance transcription by concentrating RNA polymerase II and regulatory elements. It can also be connected with chromatin remodelling and the role of enhancers acting on both strands independent of the direction of transcription.

The *AT5G58150 locus* encodes a putative, leucine-rich repeat receptor-like protein kinase. Receptor-like kinases (RLKs) form a large family of over 600 genes in the *Arabidopsis thaliana* genome [14, 15]. A large majority of RLKs contain an extracellular domain, a single-pass transmembrane domain, and a Ser/Thr kinase domain. RLK autophosphorylation appears important for signalling in plants [16]. A widespread sequence present in the extracellular part of RLKs is the leucine-rich repeat (LRR) motif, which is crucial for protein–protein interactions. The plant-specific LRR motif consists of 23–25 amino acids, which form a conserved consensus sequence: LxxLxxLxLxxNxLT/SGxIPxxLGx, where x can be any amino acid [17]. These extracellular regions assemble into a curved parallel β-sheet, forming a rod, curve, or super-helical structure. Additional N-glycosylation at asparagine residues of the LRR region, mainly at NxS/T (x ≠ P) sites, helps in the proper folding, trafficking, and performing physiological functions of RLKs. The inner surfaces of RLK extracellular structures are responsible for ligand binding or recruiting co-receptors to activate signalling pathways [18]. The LRR region is flanked by pairs of cysteines, localized about 60 amino acids from the start codon, and about 20–30 amino acids from the transmembrane domain [19]. RLKs containing the LRR motif form a large group of kinases, divided into 13 subfamilies (I–XIII) based on the organization of the extracellular domain [14]. They regulate development [17] and stress signalling [20–22] in different species by mediating plant hormone responses [23, 24] pathogen defence [25, 26] and regulating morphology and growth [27, 28]. In *Arabidopsis,* a light-regulated gene encoding a receptor protein kinase called LRRPK encoded by *AT4G29990* has been described [29]. Only recently, FERONIA (FER), a member of the *Catharanthus roseus* receptor‐like kinase 1‐like (CrRLK1L) family, has been identified as a phototropin1 substrate involved in the phototropic bending in *Arabidopsis* through modulating the activity of the H^+^‐ATPase [30]. This suggests a direct interaction between LRR kinases and blue light photoreceptors, phototropins at the plasma membrane.

AT5G58150 belongs to group VII of LRR-RLKs [14]. According to UniProt [31], it contains a putative transmembrane domain at amino acid positions 437–459. Fourteen leucine-rich-repeats (LRR) and 11 glycosylation sites are predicted to localise in the extracellular, N-terminal fragment. The C-terminus consists of a putatively inactive kinase domain. AT5G58150 is a putative plasma membrane protein. However, it was identified as part of the vacuolar proteome [32]. *Arabidopsis* screening assays suggest that AT5G58150 plays a role in salt and osmotic stress signalling [33]. In this work, we analyse the mRNA sequence and functional overlap of *AT5G58150* and *PHOT2*. We also investigate the localization, activity, and role of this RLK kinase in stress conditions in *Arabidopsis.*

## Results

### *PHOT2 *and *AT5G58150* expression profiles and sequence overlap

**Fig. 1:**
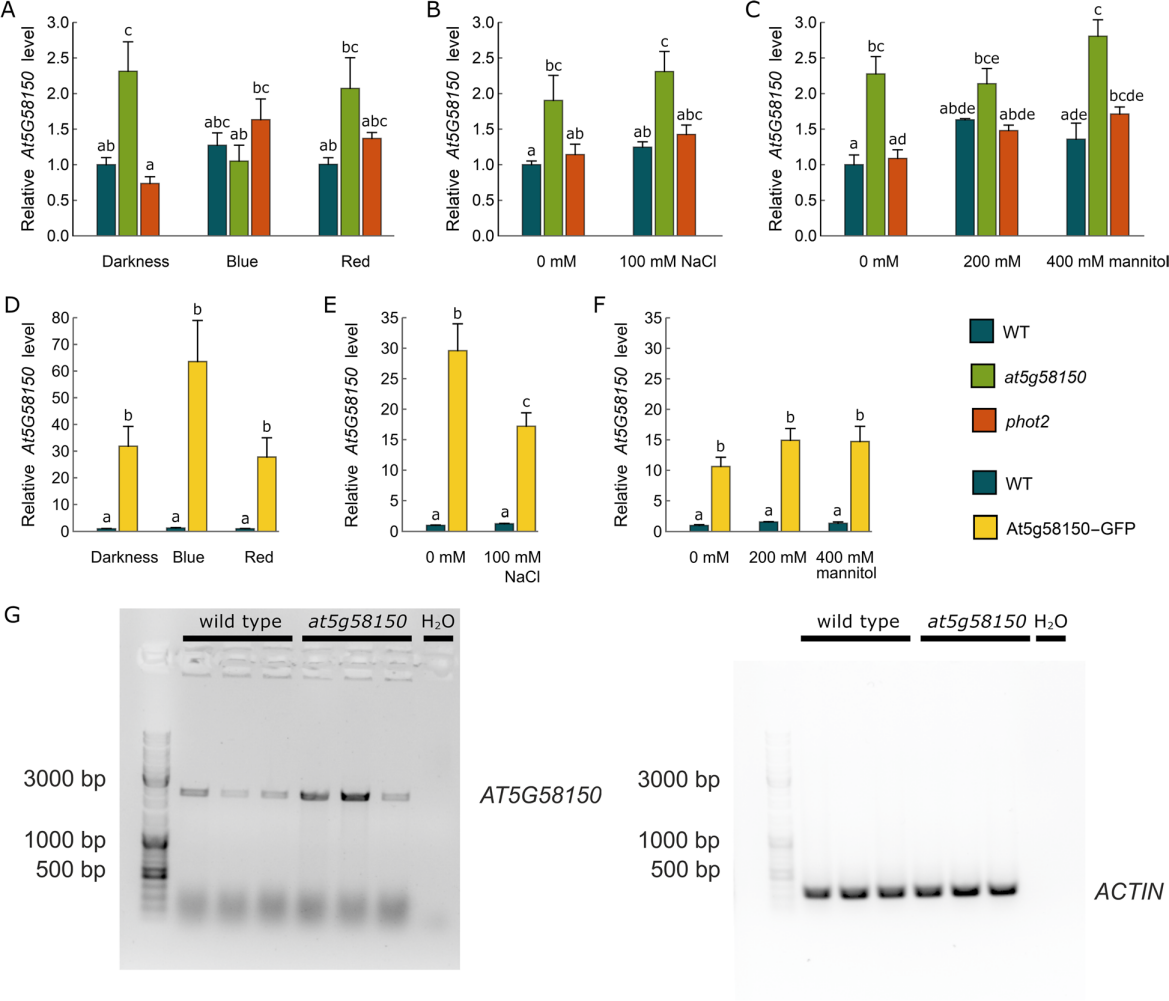
Relative expression levels of *AT5G58150* in wild-type, *at5g58150,* and *phot2* plants. **A** Dark-adapted overnight 5-week-old mature *Arabidopsis* leaves irradiated with blue or red light of 120 µmol·m^−2^·s^−1^ for 3 h or kept in darkness. **B**, **C** Seedlings were grown in vitro in the presence of 100 mM NaCl, 200 mM, and 400 mM mannitol or without any supplementation on the B5 medium for 1 week. **D**, **E**, **F** Relative expression levels of *AT5G58150* in wild-type and 35S::AT5G58150-GFP mature plants (light treatment) and seedlings (NaCl and mannitol treatment). Each bar corresponds to an average of measurements from four biological replicates. In **A**-**C** and **D**-**F**, error bars indicate the standard error. For any pair of bars, their means differ at the significance level of 0.05 if and only if they do not share a letter (tested with the Tukey method). **G** *AT5G58150* transcript levels in wild-type and *at5gs58150* plants. Actin levels served as a control of equal mRNA abundance in each lane

To investigate the impact of the putative overlap between *PHOT2* and *AT5G58150* genes*,* their expression profiles in different conditions and developmental stages were examined. We used the *phot2* mutant [34] and *at5g58150* T-DNA line, SALK_093781C, which features an insertion in the promoter region, as confirmed with the PCR method (Fig. S2). The *at5g58150* T-DNA line was previously characterized by more efficient germination when grown in the presence of 200 mM NaCl or 400 mM mannitol compared to wild-type seedlings [33]. The *PHOT2* transcript was upregulated by blue and red light in mature *Arabidopsis* leaves [35]. Thus, we examined transcript levels of *PHOT2* and *AT5G58150* in response to blue and red light (photon irradiance of 120 µmol·m^−2^·s^−1^, Fig. 1A), salt (100 mM NaCl, Fig. 1B), and osmotic (200 mM and 400 mM mannitol, Fig. 1C) stress treatments. The *AT5G58150* transcript level was affected neither by light nor salt and osmotic stress conditions in wild-type plants. The amount of *AT5G58150* mRNA was comparable in *phot2* and wild-type plants, irrespective of the treatment. However, we observed that the T-DNA line of *at5g58150* showed higher *AT5G58150* transcript levels than other plant lines almost in all investigated conditions except for blue light irradiation. The difference between wild-type and *at5g58150* was statistically significant in most of the investigated conditions. Blue light irradiation downregulated the *AT5G58150* mRNA level in the *at5g58150* line. Semi-quantitative PCR followed by gel electrophoresis confirmed the presence of the full-length *AT5G58150* transcript in the *at5g58150* line (Fig. 1G). The different levels of *AT5G58150* gene in the lanes represent the natural variability between plants, as equal amounts of RNA were taken for the experiment. The insertion in the promoter region of *AT5G58150* resulted in the upregulation of the gene expression. To investigate the role of AT5G58150, we transformed *Arabidopsis* wild-type plants with the 35S::AT5G58150-GFP construct to obtain high overexpression levels of the gene of interest and investigate its subcellular localization. The 35S::AT5G58150-GFP line showed significantly higher levels of the *AT5G58150* transcript (a ten to 20-fold increase) in all tested conditions as compared to wild-type plants (Fig. 1D, E, F). The presence of 100 mM NaCl in the medium significantly decreased the level of *AT5G58150* in the 35S::AT5G58150-GFP seedlings (Fig. 1E). No effect of the presence of mannitol was observed (Fig. 1F).

**Fig. 2:**
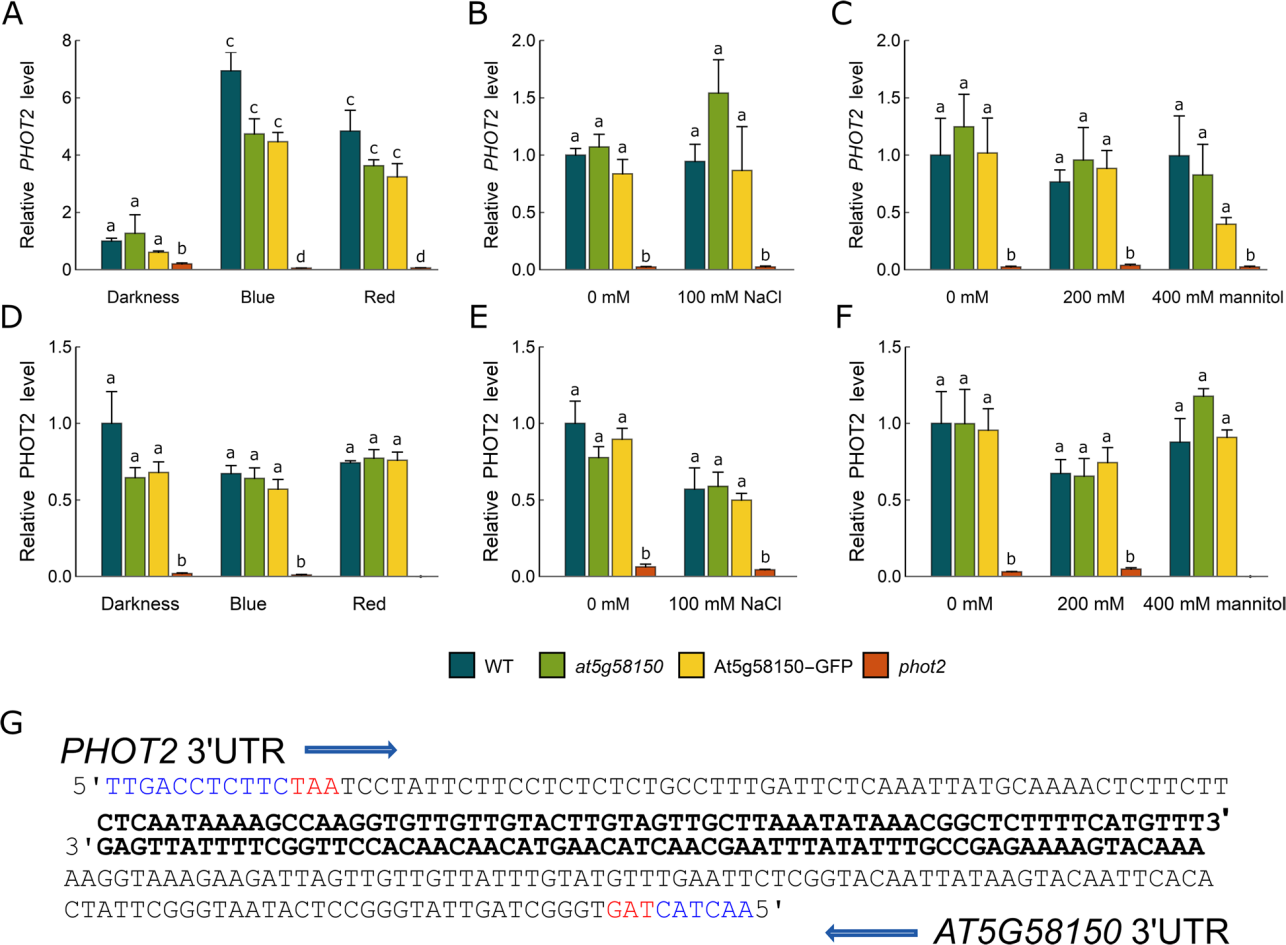
*PHOT2* transcript (**A**, **B**, **C**) and protein (**D**, **E**, **F**) relative expression levels in wild type, *at5g58150,* 35S::AT5G58150-GFP, and *phot2* plants. **A**, **D** Dark-adapted overnight, 5-week-old mature *Arabidopsis* leaves were irradiated with blue or red light of 120 µmol·m^−2^·s^−1^ for 3 h or kept in darkness. **B**, **C**, **E**, **F** Seedlings were grown in vitro in the presence of 100 mM NaCl, 200 mM, and 400 mM mannitol or without any supplementation on the B5 medium for 1 week. In **A**–**F** each bar corresponds to an average of measurements from four biological replicates. Error bars indicate the standard error. For any pair of bars, their means differ at the significance level of 0.05 if and only if they do not share a letter (tested with the Tukey method). **G** 3'UTR sequences of *PHOT2* and *AT5G58150.* Blue capital letters: coding sequences (CDS) of *PHOT2* (top) and *AT5G58150* (bottom), black: 3' UTRs of *PHOT2/AT5G58140* (top) and *AT5G58150* (bottom), bold: a 66 bp long overlap of *PHOT2* and *AT5G58150* 3'UTRs, in red: stop codons. Arrows mark the 5' to 3' direction

In parallel, we analysed the expression levels of *PHOT2* in the same plant lines and conditions as *AT5G58150* (Fig. 2 A, B, C). In line with previous data [35] *PHOT2* transcript levels were upregulated by blue and red light in wild-type *Arabidopsis* leaves. A similar upregulation was observed in *at5g58150* and 35S::AT5G58150-GFP plants. The elevation of *PHOT2* mRNA after blue and red light treatment was smaller in the *at5g58150* mutant and 35S::AT5G58150-GFP lines than in the wild-type (Fig. 2 A). *PHOT2* expression was induced neither by salt nor osmotic stress in wild-type plants, and no significant differences were observed in the *at5g58150* mutant and 35S::AT5G58150-GFP overexpressor lines (Fig. 2 B, C). Expression levels of *PHOT2* in *phot2* plants were negligible irrespective of light conditions (Fig. 2 A, B, C). We also examined PHOT2 protein levels in all lines. No significant differences were observed between the treatments and plant lines, except for the *phot2* mutant (Fig. 2D, E, F). To analyse the 3’UTR length of *PHOT2* and *AT5G58150,* we performed RACE PCR using RNA extracted from one-week-old seedlings. The 3’UTR region of *PHOT2* was much shorter than expected based on data deposited in TAIR, as it was only 119 bp long. The 3’UTR of *AT5G58150* was 169 bp and overlapped with the *PHOT2* transcript only over 66 bp (Fig. 2 G).

### AT5G58150 activity and localization

The kinase domain of AT5G58150 lacks a conserved Asp residue in the active site at position 645, which is replaced by a Glu residue, implying that the kinase may be catalytically inactive. Indeed, we obtained negative results from kinase assays using AT5G58150 recombinant kinase and histone, MBP (Myelin Basic Protein), casein, and thrumin [36, 37] as substrates (Fig. S3). The Glu residue at position 645 is conserved among Arabidopsis ecotypes, as no polymorphisms were found using the ePlant platform [38] (https://bar.utoronto.ca/eplant/).

**Fig. 3:**
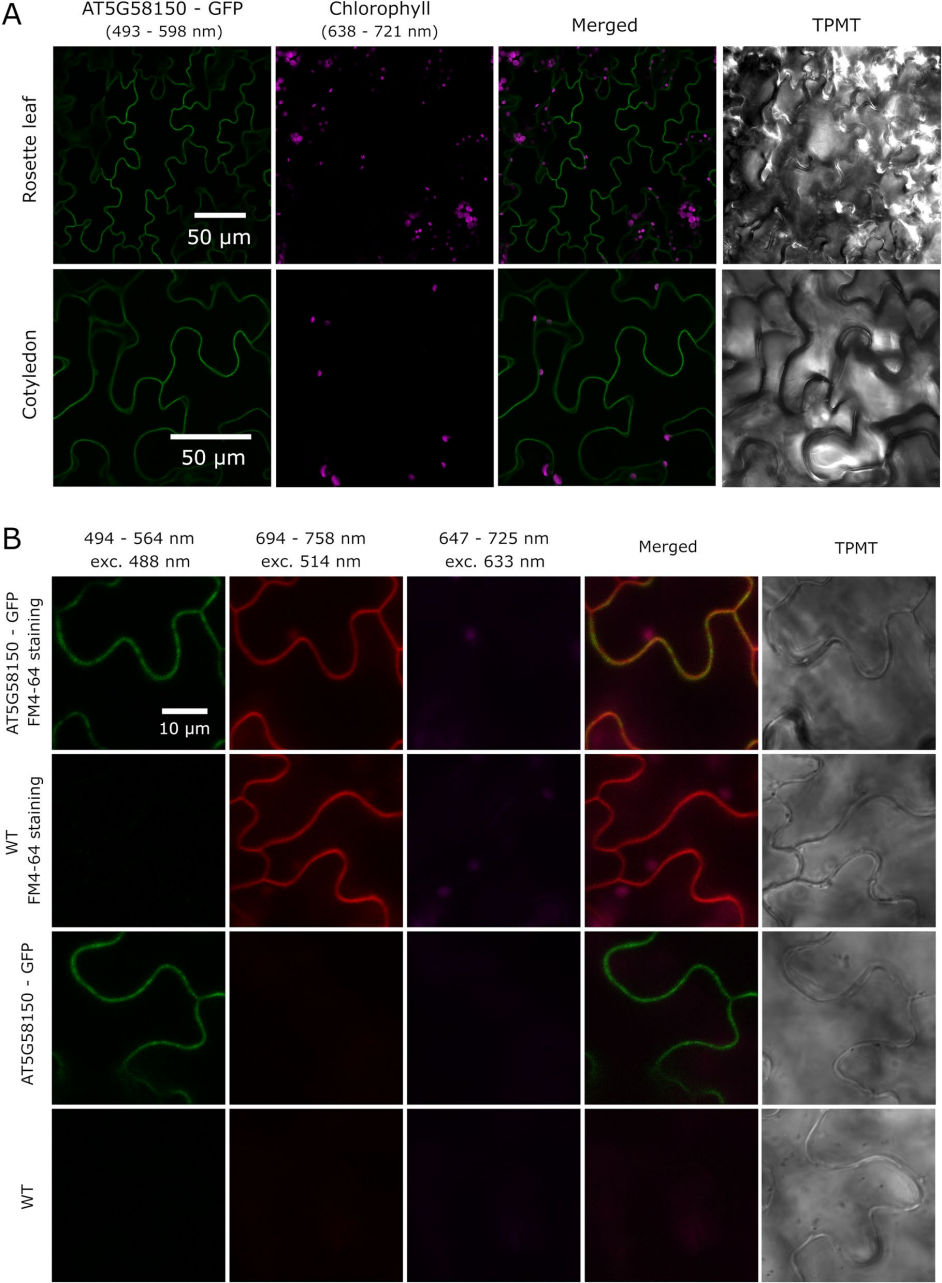
Localization of AT5G58150-GFP protein. **A** Confocal laser scanning images of *Arabidopsis* epidermal cells expressing the 35S::AT5G58150-GFP in rosette leaves of mature plants and seedling cotyledons. Chlorophyll autofluorescence is in magenta and GFP fluorescence is in green. **B** Colocalization of 35S::AT5G58150-GFP with the FM 4–64 dye in epidermal cells of rosette leaves. *Arabidopsis* WT and transgenic plants expressing 35S::AT5G58150-GFP were stained with FM 4–64. FM 4–64 fluorescence is in red, GFP fluorescence in green and chlorophyll autofluorescence in magenta. The results represent one of three independent biological replicates

AT5G58150 is predicted to bear an alpha-helical fragment, which spans the plasma membrane once. We observed GFP fluorescence at the plasma membrane in mature rosette leaves and cotyledons of the 35S::AT5G58150-GFP *Arabidopsis* line (Fig. 3 A). We also used FM 4–64 staining to further confirm the colocalization with the plasma membrane (Fig. 3B). We did not observe any GFP signal in the tonoplast, despite the identification of AT5G58150 in a survey aimed at identifying the vacuolar proteome [32]. To further analyse the localization of AT5G58150-GFP, plasma membrane, and tonoplast markers fused with mCherry [39] were transiently expressed in the *N.* *benthamiana* leaf epidermis. Only the colocalization of AT5G58150 with the plasma membrane was confirmed (Fig. S4).

**Fig. 4:**
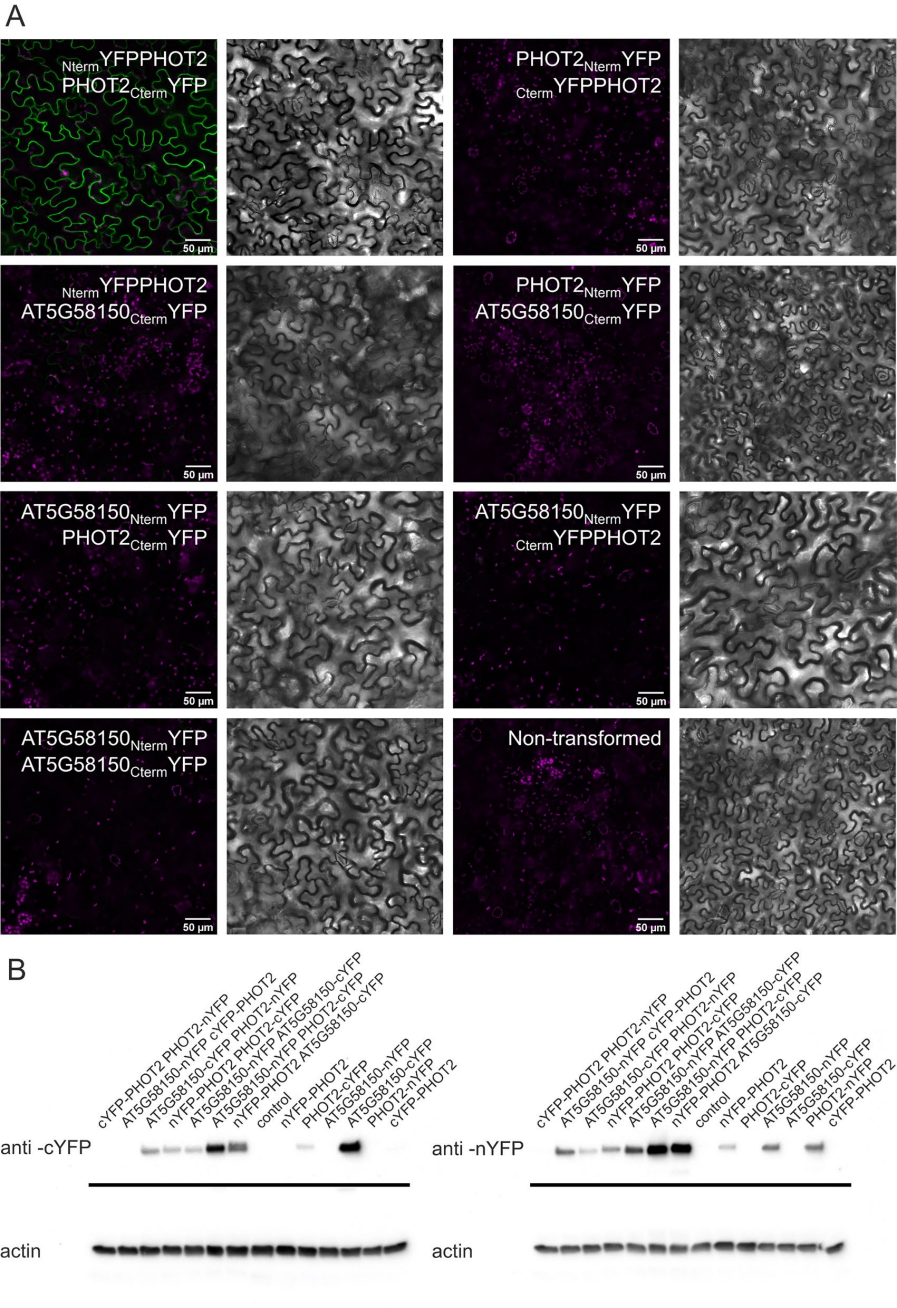
Interactions between AT5G58150 and PHOT2 examined with the Bimolecular Fluorescence Complementation Method. **A** Confocal images of *N.* *benthamiana* epidermal cells transiently co-expressing PHOT2 and AT5G58150 fused with C(N)-terminal YFP fragments in the following configurations: AT5G58150__Nterm_YFP and AT5G58150__Cterm_YFP, NtermYFP_PHOT2 and PHOT2__Cterm_YFP, _Nterm_YFP_PHOT2 and AT5G58150__Cterm_YFP, _Cterm_YFP_PHOT2 and PHOT2__Nterm_YFP, PHOT2__Cterm_YFP and AT5G58150__Nterm_YFP. Chlorophyll autofluorescence is in magenta and reconstituted YFP fluorescence is in green. The results represent one of four independent biological replicates. **B** Western Blot analysis of *N.* *benthamiana* epidermal cells transiently co-expressing PHOT2 and AT5G58150 fused with C(N)-terminal YFP fragments in the following configurations: AT5G58150__Nterm_YFP and AT5G58150__Cterm_YFP, _Nterm_YFP_PHOT2 and PHOT2__Cterm_YFP, _Nterm_YFP_PHOT2 and AT5G58150__Cterm_YFP, _Cterm_YFP_PHOT2 and PHOT2__Nterm_YFP, PHOT2__Cterm_YFP and AT5G58150__Nterm_YFP, AT5G58150__Nterm_YFP, AT5G58150__Cterm_YFP, _Nterm_YFP_PHOT2, _Cterm_YFP_PHOT2, PHOT2__Nterm_YFP, PHOT2__Cterm_YFP probed with anti-cYFP and anti-nYFP. The lower image shows the same blot after stripping and probing with anti-actin antibodies. Uncropped image in Fig. S5. The results represent one of three independent biological replicates

RLK signalling is often triggered by protein interactions in the extracellular domain [14]. Thus, we tested whether AT5G58150 forms homodimers, using the Bimolecular Fluorescence Complementation (BiFC) assay (Fig. 4 A). No fluorescence was detected in the case of AT5G58150 C-terminal fusions with YFP fragments, despite expression levels sufficient to be detected by a Western Blot using antibodies recognizing N- and C-terminal YFP fragments, respectively (Fig. 4B, Fig. S5). Phot2 is a plasma membrane-bound kinase, with only a few interacting proteins identified [40, 41]. Thus, we tested whether AT5G58150 interacts with PHOT2 at the plasma membrane using the BiFC method (Fig. 4). For the positive control, leaves were co-transformed with _Nterm_YFP-PHOT2 and PHOT2-_Cterm_YFP vectors and a strong fluorescent signal indicative of phot2 dimerization was observed, as reported previously [42]. No fluorescent signal was observed in plants co-transformed with _Nterm_YFP_PHOT2 and AT5G58150__Cterm_YFP, despite expression detectable by Western Blot (Fig. 4 A, Fig. S5). This result is consistent with the absence of AT5G58150 dimerization. The level of the PHOT2__Cterm_YFP protein was very low when co-expressed with _Nterm_YFP_PHOT2 and especially AT5G58150__Nterm_YFP. _Cterm_YFP_PHOT2 construct showed very weak expression (Fig. S5). In contrast to BiFC results, weak interaction between the N-terminal part of PHOT2, containing LOV domains, and the kinase domain of AT5G58150 was observed in the split-ubiquitin-based membrane yeast two-hybrid system in one of the bait and pray configurations, regardless of the light conditions. No interaction of the AT5G58150 kinase with full-length phototropin2 was detected (Fig. S6).

We also investigated if higher expression levels of AT5G58150 in *at5g58150* insertion and 35S::AT5G58150-GFP lines have an impact on chloroplast avoidance, which is controlled by phot2 upon high-intensity blue light treatment [34, 43]. No significant differences in chloroplast responses to continuous blue light irradiation were observed (Fig. S7).

### The role of AT5G58150 in salt and osmotic stress tolerance

**Fig. 5:**
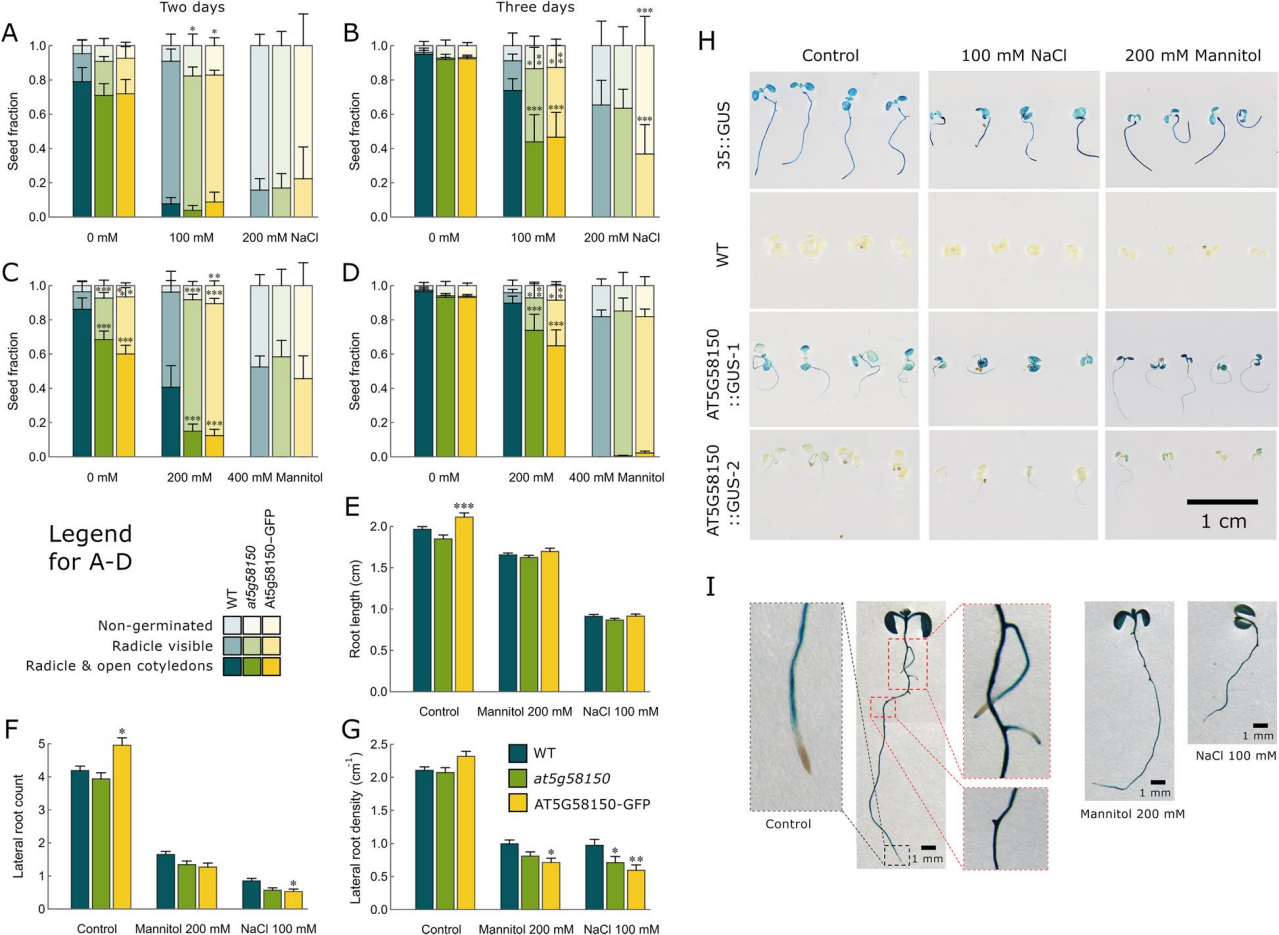
The impact of salt and osmotic stress on the growth of wild type, *at5g58150,* and 35S::AT5G58150-GFP seedlings. **A**, **B** Germination of seeds sown on media with either 100 mM NaCl or 200 mM NaCl or with no additional solute. The experiment was repeated 5 times, with 52 seeds per group. **C**, **D** Germination of seeds sown on media with either 200 mM mannitol or 400 mM mannitol or without any component added. The experiment was repeated seven times, with 52 seeds per group. In **A**–**D**, seeds were classified into three groups: (i) non-germinated, (ii) with visible radicle and cotyledons, if visible, unopened, (iii) with radicle visible and opened cotyledons, on the second (**A**, **B**) and third (**C**, **D**) day of growth. Error bars represent SE. Asterisks mark groups that differ in log-odds ratios from wild-type control grown on the same medium (likelihood ratio χ^2^ test, *p = 0.01–0.05; **p = 0.001–0.01, ***p < 0.001, adjusted with Hommel’s method). **E**-**G** Primary root length (**E**), lateral root number (**F**) and lateral root density (**G**) of 9-day-old seedlings growing on the media with 100 mM NaCl or 200 mM mannitol or no solute added. Lateral root density was calculated as the number of lateral roots per cm of the primary root. The experiment was repeated 3 times, with 48 seeds per group. Asterisks mark groups that differ in mean from wild-type control grown on the same medium (*p = 0.01–0.05; **p = 0.001–0.01, ***p < 0.001, adjusted with Hommel’s method). **H** The impact of salt and osmotic stresses on the promoter activity of 35S::GUS*,* and *AT5G58150*::GUS seedlings. Seeds were sown on media either with 100 mM NaCl or 200 mM mannitol or without added components. After 7 days, the promoter activity was visualised with GUS staining. Each experiment was repeated five times, with 52 seeds sown per group. **I** *AT5G58150* expression pattern in seedlings grown in control conditions (left picture) and the presence of 200 mM mannitol (middle picture) or 100 mM NaCl (left) added to the growth media after 8 days of germination. The experiment was repeated three times

LRR-RLK kinases are known to play an essential role in salt and osmotic stress responses in *Arabidopsis* [33] and rice [44]. To examine the impact of salt stress on the germination and growth of wild-type, *at5g58150,* and 35S::AT5G58150-GFP seedlings, seeds were sown on media with 100 mM NaCl, 200 mM NaCl or without any additional solute added (Fig. 5 A, B). Two and three days after transferring the plates from the cold room to the culture chamber, the seedlings were counted and classified into three groups: (i) non-germinated, (ii) with visible radicle and cotyledons, if visible, unopened, (iii) with radicle visible and opened cotyledons. As examined with ANOVA and posthoc tests (details in Supplement 2 with Supporting Tables S1 – S4), both the presence of salt and overexpression of AT5G58150 led to a significant delay in seedling development (Fig. 5A, B). The interaction between these two factors was substantial and statistically significant, indicating that the effects of salt stress and AT5G58150 overexpression are synergistic. The differences between lines were more pronounced 3 days after the end of cold treatment (Fig. 5B). In the presence of 100 mM NaCl, the fraction of seedlings with both radicles visible and open cotyledons was significantly lower in 35S::AT5G58150-GFP and *at5g58150* lines than in the wild-type, while the proportion of seedlings with only radicles released from the seed coat was higher. Supplementation of the medium with 200 mM NaCl led to a greater delay in seedling development. No seedlings grown on 200 mM NaCl released cotyledons in any of the studied lines. The fraction of three-day-old seedlings with visible radicles, but cotyledons unopened, was significantly lower for the 35S::AT5G58150-GFP line than in wild-type plants, while the proportion of non-germinated seeds was greater in this line (Fig. 5B). To examine the impact of osmotic stress in the absence of ionic stress, the germination of wild-type, *at5g58150,* and 35S::AT5G58150-GFP seeds on media with either 0 mM, 200 mM, or 400 mM mannitol was investigated (Fig. 5C, D). Like in the case of salt stress, the presence of mannitol delayed seedling development. There was a significant interaction (Supporting Tables S1 – S3 in Supplement 2) between the effect of mannitol and the effect of overexpression of AT5G58150. The fraction of two-day- and three-day-old seedlings with radicles visible and open cotyledons was significantly lower in the 35S::AT5G58150-GFP and *at5g58150* lines than in the wild-type when 200 mM mannitol was added. Correspondingly, a greater fraction of 35S::AT5G58150-GFP and *at5g58150* seeds either did not germinate or produced seedlings at the early stage of development, with radicles visible. The presence of 400 mM mannitol delayed seedling development to a greater extent, but the differences between plant lines were not significant (Fig. 5C, D).

To investigate the activity of the putative promoter sequence in salt and osmotic stress conditions, we created two *AT5G58150*::GUS *Arabidopsis* lines. According to [45], the promoter of *AT5G58150* is active in all *Arabidopsis* organs at a moderately high level. Our lines differed in the level of GUS activity (Fig. 5H). However, seedlings of both *AT5G58150*::GUS lines did not show substantial differences in the activity of GUS in the presence of 200 mM mannitol or 100 mM NaCl compared with control conditions (Fig. 5H). A closer examination of seedlings revealed that *the AT5G58150* promoter is active in almost the entire seedling except for the apical meristem of the primary root and elongation zone of lateral roots (Fig. 5I). In the primary root, the expression of *AT5G58150*, marked with its promoter activity, started in the differentiation zone. In developed lateral roots, it started in the differentiation zone, but the *AT5G58150* promoter activity was also high in the meristematic zone and very high in lateral root primordia. The tested promoter was active in all cotyledon tissues (Fig. 5I).

The expression pattern of *AT5G58150* indicates that this gene may play a role in the development of root system architecture (Fig. 5I). Therefore, we measured the primary root length of 9-day-old wild-type, *at5g58150* and 35S::AT5G58150-GFP seedlings grown on the control medium and media supplemented with either 200 mM mannitol or 100 mM NaCl (Fig. 5 E). In control conditions, the 35S::AT5G58150-GFP line developed significantly longer primary roots than the wild-type plants (statistical analysis in Supplement 2 with Supporting Table S4). The presence of NaCl and, to a smaller extent, of mannitol reduced the primary root length in all examined lines. The decrease due to salt and osmotic stress was greater for 35S::AT5G58150-GFP than for wild-type plants. In control conditions, 35S::AT5G58150-GFP produced significantly more lateral roots than the wild type, while the opposite differences were observed on the medium with NaCl added (Fig. 5F). As expected, the presence of mannitol and NaCl severely decreased lateral root density (Fig. 5G). The reduction in lateral root density due to the osmotic and salt stress was significantly greater for 35S::AT5G58150-GFP seedlings than for wild-type plants, as tested with the interaction contrasts (Supplement 2 with Supporting Table S5). The presence of 100 mM NaCl reduced lateral root density by around 54%, and 74% for wild type and 35S::AT5G58150-GFP, respectively. A similar, though smaller, reduction was caused by the presence of 200 mM mannitol. The lateral root density of 35S::AT5G58150-GFP seedlings was significantly lower on media with either NaCl or mannitol, but not on the control medium (Fig. 5G). The second overexpressing line, *at5g58150*, also exhibited lower lateral root density than the wild-type in the presence of solutes in the medium, though the difference was significant only in the presence of 100 mM NaCl. These results may indicate that overexpression of AT5G58150 leads to higher plant sensitivity to salt and osmotic stress and that AT5G58150 may be involved in the regulation of root system development under osmotic and salt stress conditions.

### The AT5G58150 interaction network

**Fig. 6:**
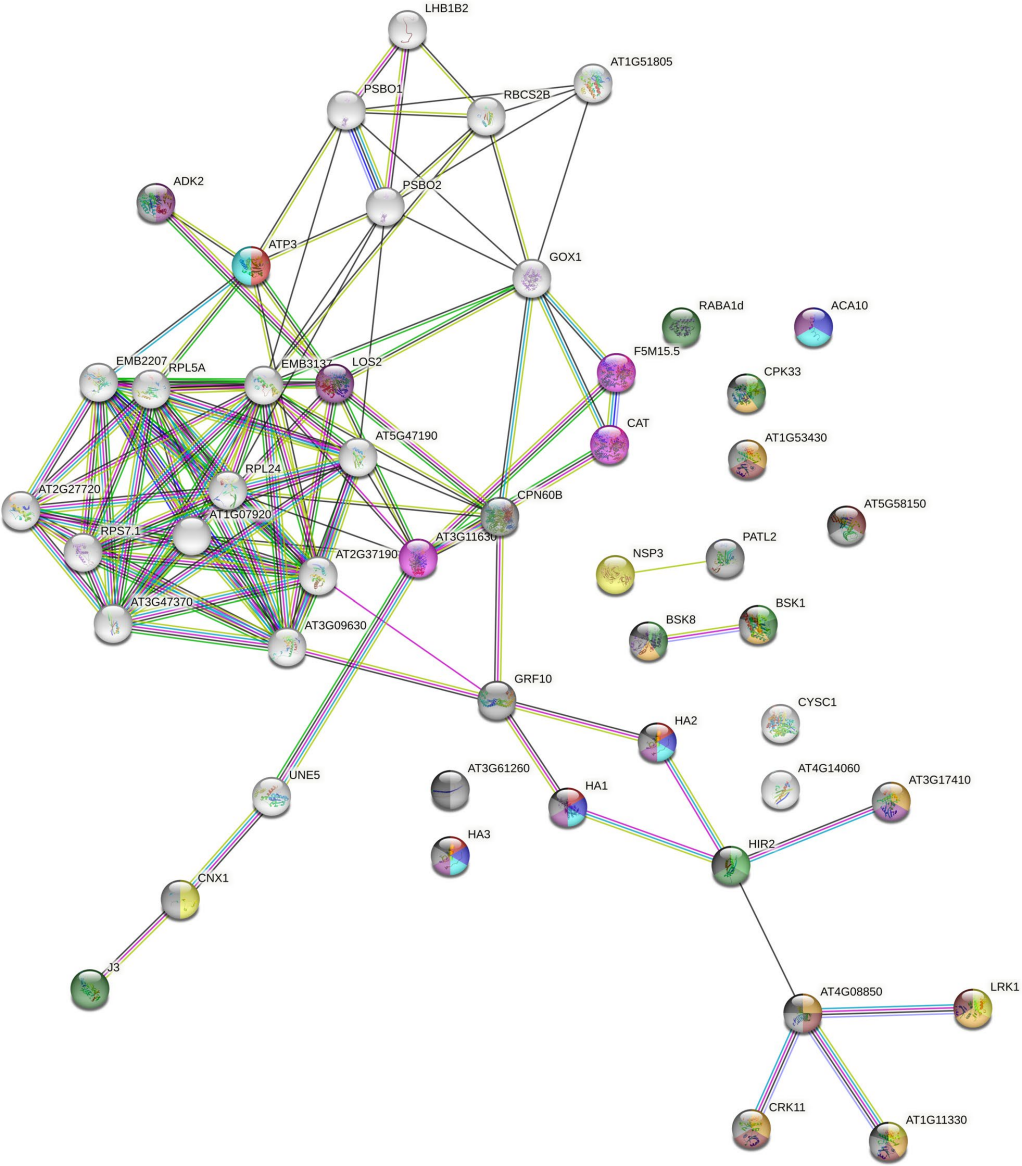
Protein interaction network generated with String [48] (https://string-db.org/) based on the results of Mass Spectrometry analysis of proteins that co-immunoprecipitated with GFP-tagged ATAT5G58150. Functional colour coding based on Annotated Keywords to proteins from UniProt (see Table 1)

To elucidate the biological function of AT5G58150, protein immunoprecipitation followed by Mass Spectrometry (MS) analysis was performed from 8-day-old seedlings expressing 35S::AT5G58150-GFP. A 35S::GFP expressing line served as a control for the experiments. A list of 49 proteins fulfilling the interactome criteria, specified in the Methods section, has been chosen as possible candidates for the functional network of AT5G58150 (Fig. 6, Table 1), with the bait protein being predominant. The list of all proteins from the MS analysis, which fulfill the interaction criteria, together with the values of parameters taken into account in the calculations, is given in Supporting Table S6 of Supplement 1. Proteins identified by MS fall into three main categories: ribosomal, components of the photosynthetic apparatus, and membrane-bound. Due to the confirmed localization of the AT5G58150-GFP protein (Fig. 3, Fig. S4), further analysis focused on proteins that localize to the plasma membrane. Those proteins form a network, which divides into clusters sharing (i) a protein kinase activity, combined with lectin binding; (ii) hydrogen ion/ion transport, ATPase, and translocase activities (iii) lipoproteins combined with mirystolase activities, (iv) brassinosteroid pathway proteins, and (v) peroxidases. Interestingly, three putative members of the LRR RLK family were identified, AT1G51805, AT1G53430, and AT4G08850. Additionally, seven more of the identified proteins exhibit serine-threonine kinase activity. HA2 [46] and CYSC1 [47] are responsible for root hair development. The list of all proteins from the MS analysis, with their functional descriptions from the String database [48], is given in Supplement 1 with Supporting Table S7.

**Table 1:**
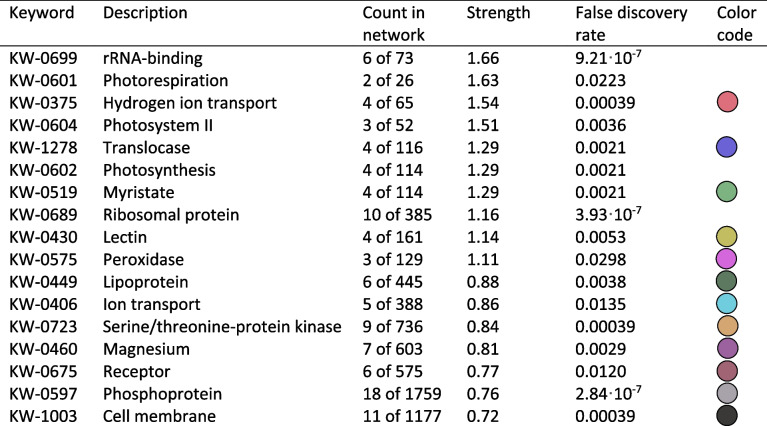
Functional classes of proteins co-immunoprecipitated with AT5G58150 in the network shown in Fig. 6 and their colour codes

## Discussion

The promoter of the *AT5G58150* gene is predicted to contain light-responsive cis-elements (LREs) and sequences over-represented in light-induced promoters (SORLIP1 and SORLIP2) (AtcisDB database, [49]). Nevertheless, no influence of light on transcript levels of *AT5G58150* was observed in wild-type plants (Fig. 1A). The disruption of genome integrity in the sequence upstream of the start codon of the *AT5G58150* gene leads to the lower abundance of the transcript upon blue light treatment (Fig. 1A). It might be caused by the disturbance of blue, but not red-light-dependent regulation of *AT5G58150* expression. The observed NaCl-induced decrease in the level of *AT5G58150* in the 35S::AT5G58150-GFP line may indicate posttranscriptional control of the transcript accumulation, as reported for several salt stress-related genes [50].

The *PHOT2* and *At5G58150* genes are localized tail to tail on the complementary DNA strands. The results of the 3’-RACE analysis showed a relatively short overlap in the 3’UTRs of those genes, comprising only 66 bp (Fig. 2G). This is substantially different from data deposited in the TAIR database for the 3'UTR of *PHOT*2. cis-NATs can influence the corresponding mRNA levels by regulation of transcription or post-transcriptional events, including transcript stability. They can also control the protein levels by impacting translational efficiency. No direct impact of the presence of *AT5G58150* and *PHOT2* transcripts on their mutual expression was observed. The upregulation of *PHOT2* mRNA by light was not connected to a decrease in *AT5G58150* abundance in wild-type (compare Fig. 1A and Fig. 2A). The elevated levels of *AT5G58150* transcript in the 35S::AT5G58150-GFP line (Fig. 1 D, E, F) had no impact on either mRNA or protein levels of phototropin2 (Fig. 2). These results point to the lack of the mutual regulation of these gene products under the tested conditions.

Investigation of localization of AT5G58150 fused to GFP, using stable and transient transformation assays, indicated that it is a plasma membrane protein (Fig. 3 and Fig. S4). We performed further colocalization studies to investigate whether AT5G58150 is additionally targeted to the vacuolar membrane, as it was identified in the analysis of the vacuolar proteome [32]. AT5G58150-GFP colocalized with the plasma membrane stained with FM 4-64 (Fig. 3B). It is absent from the mCherry-tagged tonoplast. This is seen in areas around the nucleus and along the vacuolar strands, where the tonoplast is far from the cell membrane (Fig. S4). To elucidate the function of AT5G58150 at the plasma membrane, we investigated its ability to form dimers. LRR RLK family members often form complexes that serve as primary signalling units, such as BOTRYTIS INDUCED KINASE1 (BRI1), BRI1‐ASSOCIATED KINASE1 (BAK1), and BRI1 KINASE INHIBITOR1 (BKI1) [51]. We did not observe homodimer formation for AT5G58150 using the BiFC method (Fig. 4). We also investigated whether PHOT2 may be a partner for AT5G58150 at the plasma membrane, but no interaction was detected or it was too weak to be physiologically relevant (Fig. 4, Fig. S6). Our results show that AT5G58150 is not involved in phototropin2 signalling to elicit chloroplast movements. However, AT5G58150 probably requires a protein interactor, as it lacked detectable kinase activity due to an Asp to Glu substitution at position 645 (Fig. S3). RLK defective kinases are relatively common in the *Arabidopsis* genome. Interactions through the inactive kinase domains with other proteins may be important for signal transduction by phosphorylation-independent mechanisms. Examples of such kinases include MARK (maize atypical receptor kinase) or *Arabidopsis* SUB (STRUBBELIG) [16].

Our results showed that AT5G58150 overexpression did not enhance the growth of *Arabidopsis* seedlings in the presence of NaCl or mannitol. Salt and osmotic stress resistance were reported previously for the same *at5g58150* SALK line as used in this study [33]. Both *at5g58150* and 35S::AT5G58150-GFP lines were characterized by higher expression levels of the *AT5G58150* gene (Fig. 2), and yet they were more susceptible when grown in the presence of NaCl or mannitol (Fig. 5). A detailed investigation of *AT5G58150*::GUS seedlings and measurements of primary root and lateral root density indicated a putative role for AT5G58150 in the regulation of root system architecture. Overexpression of AT5G58150 amplified the reduction of lateral root production in the presence of mannitol or NaCl (Fig. 5 F-G). Significant impairment in lateral root production in the presence of NaCl was previously reported for mutants of genes involved in response to salt stress such as *SOS3* [52] and *SnRK2.10* [53]. Thus, growth retardation observed in salt or osmotic stress conditions may stem indirectly from the functions played by AT5G58150 in lateral root development. AT5G58150 belongs to group VII of LRR-RLKs [14]. This family contains 10 members, including inactive kinases, MUSTACHES (MUS) and MUSTACHES-LIKE (MUL), which redundantly control lateral root formation via regulation of cell wall remodeling enzymes at the first stage of lateral root formation [54]. Interestingly, *MUS*, *MUL* and *AT5G58150* show similar expression pattern in primordia. Further experiments are necessary to investigate if AT5G58150 acts together with MUS and/or MUL in lateral root formation.

The Mass Spectrometry analysis of immunoprecipitated proteins suggests that the AT5G58150 protein may interact with several kinases, including some involved in brassinosteroid signalling. BSK1 and BSK8 (Brassinosteroid-Signalling Kinases) play highly redundant roles [55, 56]. These proteins are direct phosphorylation targets of BRI1, thus acting downstream of this protein in the brassinosteroid pathway [56]. Another important group of putative interactors was plasma membrane-localized H^+^-ATPases, namely HA1, HA2, and HA3. The HA1 and HA2 double mutant is embryo-lethal, indicating their strict functional cooperation [57]. Both proteins interact with the BRI-associated receptor kinase 1 (BAK1), by forming a complex at the plasma membrane [58]. As previously mentioned, BAK1 together with BRI1 forms a well-characterized signalling unit [51], and BRI1, interacts with HA1 [59]. Thus, AT5G58150 may be a new component of the brassinosteroid signalling pathway. The proton gradient generated by H^+^-ATPases results in external acidification and/or internal alkalinization of the cell and mediates growth responses through the acid growth mechanism [60]. This observation, together with the putative involvement in brassinosteroid signalling [61] is in line with our findings that AT5G58150 may be involved in the development of the root system architecture. Moreover, a BLAST search indicated that AT5G58150 shows homology with BRI1-Like (BRL1) receptor kinase (E-value of 7 10^–89^, percent identity 30.65%) and BRI1 Like 3 (BRL3) (E-value of 1·10^–88^, percent identity 31.51%) proteins [62]. BRL1 and BRL3 show similar expression patterns in the inner root tissues, the endodermis and pericycle, [63], as AT5G58150 (Fig. 5I, eFP Browser [64]). BRL3 forms a complex regulating root development with BSK1, BSK3, BAC1, FERONIA and HA2 ATPase 2 [65]. Some of those proteins (BSK1, HA2) were identified in our co-immunoprecipitation study of AT5G58150, further suggesting its involvement in brassinosteroid signalling. We examined genes co-expressed with *AT5G58150* in publicly available data, using the Arabidopsis Co-expression Tool [66, 67], and found genes of the brassinosteroid pathway, involved in root development as well as cell wall and junction formation. The results of the biological term enrichments analysis are collected in Table S9. Using the Athena tool (https://athena.proteomics.wzw.tum.de/master_arabidopsisshiny/), we found that AT5G58150 co-expresses with components of the brassinosteroid pathway both at the proteomic (Fig. S8) and transcriptomic (Fig. S9) levels. The proteins identified with Mass Spectrometry should be treated as candidate partners of AT5G58150 and targets of detailed, confirmatory interactions assays, performed with complementary techniques. Such future studies will allow to better explain the function of AT5G58150 in roots. To fully elucidate the function of *AT5G58150*, analysis of the loss of function mutants is also needed.

## Conclusions

AT5G58150 is a membrane protein, with an inactive kinase activity, whose biological function may be linked to the proper root formation under salt and osmotic stress.

## Methods

### Plants and growth conditions

The *phot2* (*npl1-1* mutant) was kindly gifted by J.A. Jarillo [34]. Wild type Col-0 and *at5g58150 SALK_093781C* [33] were purchased from Nottingham Arabidopsis Stock Centre. Line homozygosity was confirmed with Phire Plant Direct PCR Master Mix (Thermo Fisher Scientific). The 35S::AT5G58150-GFP (vector pK7FWG2) and AT5G58150prom::GUS (vector pORE-R1), 35S::GFP [68], vector pGreenII0029) lines were prepared in the Col-0 background using the floral dip method and screened for kanamycin resistance. Primer sequences used for cloning and genotyping are given in Table S8. Seeds sown in Jiffy-7 pots (Jiffy Products International AS) were left at 4 °C for 2 days. Subsequently, plants were transferred to a growth chamber (Sanyo MLR 350H) at 23 °C, 80% relative humidity, with a photoperiod of 10 h light and 14 h darkness, at 70 μmol·m^−2^·s^−1^ of light supplied by fluorescent lamps (FL40SS.W/37, Sanyo, Japan). Five-week-old plants were used in experiments with leaves. In experiments with seedlings, seeds were sterilized with 70% ethanol for 5 min, 50% commercial bleach for 5 min, washed three times in distilled, sterile water, and sown on a solid B5 medium with vitamins and 1% sucrose.

### RACE PCR

To determine the 3' ends of *PHOT2* and *At5g58150* transcripts, 7-day-old seedlings grown in a short-day photoperiod were dark-adapted overnight. Total RNA was extracted using Spectrum Plant Total RNA Kit (Sigma-Aldrich). DNA contaminations were removed with DNase I (Sigma-Aldrich) during purification on columns. RACE-ready cDNA was prepared using the GeneRacer Kit (Invitrogen) following the manufacturer's protocol. Decapped mRNA with GeneRace RNA Oligo, ligated at the 3' end, served as a template for PCR with Platinum Taq DNA Polymerase High Fidelity (Thermo Fisher Scientific). 3' ends of *PHOT2* and *At5g58150* mRNAs were amplified with GeneRace 3' and *PHOT2* specific primer (5'-CGGACTTGGACATTGACCTC-3') and *AT5G58150* specific primer (5'-TGGGTGAGAGGGTTGGTGAGGCAGGGAC-3'), respectively. PCR products were cloned into the pGEM-T Easy vector (Promega) and sequenced.

### Treatments

To evaluate the effects of AT5G58150 overexpression in the presence of salt and osmotic stresses, seeds were sown on the B5 media with vitamins, 1% sucrose (control), either without any additional solutes or with 100 mM NaCl, 200 mM NaCl, 200 mM mannitol or 400 mM mannitol. After sowing, plates were kept in a cold room for two days and transferred to the growth chamber. After two or three days of germination, seeds, and seedlings were counted and classified into three groups: (i) non-germinated seeds, (ii) seedlings with visible radicle and cotyledons, if visible, unopened, and (iii) seedlings with visible radicle and open cotyledons. To analyse the root architecture, 9-day-old seedlings growing on vertical plates were imaged with a scanner together with a scale bar. The root length was measured from the root-shoot junction to the root tip with ImageJ [69]. The emerged lateral roots of 9-day-old seedlings were counted. Lateral root density was calculated as the number of lateral roots per cm of the primary root. For the analysis of the effect of salt and osmotic stress on gene expression, seedlings were grown for 7 days and then frozen in liquid nitrogen (50 plants per each group in four biological replicates).

To examine the effect of light on transcript levels, dark-adapted overnight 4-week-old soil-grown *Arabidopsis* plants were irradiated with blue (450 nm) or red light (655 nm) of 120 µmol·m^−2^·s^−1^ (Lumileds Lighting LEDs, US) for 3 h. Light-treated leaves were collected simultaneously with those kept in darkness as a control. The experiment was performed in four independent biological replicates. Each sample contained the leaves of two plants growing in the same Jiffy pot. Samples were frozen in liquid nitrogen immediately after treatments. Chloroplast movements were assessed by the photometric method, as described in [70], and analysed using a custom-written Mathematica script (Wolfram Research, US).

### Expression analysis

RNA isolation and real-time PCR were performed as in [35], except that oligo(dT) primers were used for RNA reverse transcription. Primer sequences had been listed for *PHOT2* in [35] for reference genes: *UBC, PDF2,* and *SAND* in [71] for *AT5G58150* in Table S8. Each sample was run in three technical replicates. The relative expression of each gene in a sample was determined using the mean value of *Ct* for all samples. Expression levels were normalized using factors calculated by geNorm v3.4 [72]. Actin primers used for *AT5G58150* level analysis using PCR followed by 1% agarose gel electrophoresis are listed in Table S8.

Proteins were extracted according to [7]. Samples were homogenized, weighed, and adjusted to an equal mass. SDS-PAGE was performed on 7.5% polyacrylamide gels followed by a semi-dry protein transfer (Biorad). Western Blot was performed as described in [42]. Anti-PHOT2 (AS10 721) antibodies, used at a dilution of 1:5000, were obtained from Agrisera [73], Anti-cYFP antibodies (AS111775, Agrisera) at a dilution of 1:10,000 and anti-nGFP antibodies to detect nYFP (632,375 Living Colors GFP Monoclonal Antibody Clontech) at a dilution of 1:10,000. Intensities of the signals in each sample were normalized to actin levels (determined by anti-actin antibodies, AS132640, Agrisera, diluted at 1:2500). A chemiluminescent signal was detected with the BioSpectrum Imaging System (UVP Ultra-Violet Products Ltd). Densitometric quantification was performed using ImageJ.

### Laser scanning confocal microscopy and bimolecular fluorescence complementation (BiFC)

Fluorescent-protein tagged constructs for microscopy studies were prepared using the MultiSite Gateway system (Invitrogen). The *PHOT2* construct was described in [42]. The *AT5G58150* gene was cloned into pDONR221 with Easy-A High Fidelity polymerase (Strategene) using primers listed in Supporting Table S8 and confirmed by sequencing. The pK7FWG2 vector was used for localization studies to obtain a C-terminal GFP fusion. Transient transformation of *Nicotiana benthamiana* leaves was performed according to [74]. Microscopic observations were performed using the Axio Observer.Z1 inverted microscope (Carl Zeiss, Jena, Germany) equipped with the LSM 880 confocal module.

*Colocalization with plasma membrane*: To visualize the plasma membrane, FM 4-64 (SynaptoRed) was used at a final concentration of 10 μg/ml. Rosette leaves of *A. thaliana* expressing 35S::AT5G58150-GFP were visualized after 20 min of incubation with the dye using the Plan-Neofluar 40x/NA 1.3 oil immersion objective. Three emission channels were recorded, to capture fluorescence emitted by GFP (using excitation 488 nm, emission 493–598 nm), FM 4-64 (514 nm excitation, 694–758 nm emission), and chlorophyll (633 nm excitation, 647–725 nm). Vectors expressing plasma membrane and tonoplast markers fused with mCherry [39] were co-expressed with AT5G58150-GFP in *Nicotiana benthamiana* leaves. The lower epidermis of transformed leaves was observed with the 40 × objective. Three fluorescence channels were recorded (GFP: 488 nm excitation, 647–721 nm emission; mCherry 561 nm excitation, 582–661 nm emission; chlorophyll 633 nm excitation, 647–721 nm emission).

*Bimolecular colocalization assay*: pSITE vectors were used to prepare constructs for BiFC analysis [75]. Microscopic observations of the lower epidermis of *N. benthamiana* leaves were performed two days after transformation, using Plan-Apochromat 20x/NA 0.8 dry objective. YFP fluorescence was excited by the 514 nm line of the Ar laser and collected in the range of 519–620 nm, chlorophyll was excited by the 633 nm He–Ne laser in the range of 647–721 nm.

### Kinase assays

The AT5G58150 kinase domain (amino acids 491–785) was cloned into the pGEX4T-1 vector using the BamHI and SmaI sites and sequenced. The primer sequences are listed in Table S8. The recombinant kinase was expressed in the *E.coli* BL21 strain and affinity purified using glutathione sepharose. For the kinase activity test, the purified protein (2µg) was incubated with 10 μg of a substrate and with 50 μM ATP supplemented with 1 μCi of [γ-^32^P]ATP in the kinase buffer (25 mM Tris–HCl, pH 7.5, 5 mM EGTA, 1 mM DTT, 30 mM MgCl_2_, total reaction volume 25 μl) at 30 °C for 30 min. Kinase activity assays were tested using histone (H-9250, Sigma), MBP (Myelin Basic Protein, M1891, Sigma), β-casein dephosphorylated (C-8157, Sigma), and thrumin (purified as a GST fusion from vector pGEX6T1) as substrates.

### Split-ubiquitin-based membrane yeast two-hybrid (MYTH) system

Protein interactions in yeasts were tested with the split-ubiquitin-based MYTH system (MoBiTec), modified by the introduction of Gateway cloning sequences [76]. Bait and prey vectors with full-length phototropin2, its N- (amino acids 1–572), or C-terminal domains (amino acids 573–915) were described in [42]. The kinase domain of the *AT5G58150* gene from pDONR221 was cloned into pPR3_N_Gateway prey and pDHB1_Gateway bait vectors. Yeast transformation and handling were described in [76]. For scoring interactions, transformed yeasts plated on agar plates were kept at 30 °C either in darkness or under blue light (~ 20 μmol·m^−2^·s^−1^, 450 nm) for 4 days. Each experiment was repeated three times.

### Promoter activity studies

The *AT5G58150* promoter sequence (-1368 to -1, including the 5’UTR, according to the AGRIS, http://arabidopsis.med.ohio-state.edu) was cloned into the pORE-R1 plasmid using the HindIII and EcoRI sites. Primer sequences used for cloning are listed in Table S8. C58 *Agrobacterium tumefaciens* strain was used to transform wild-type Col *Arabidopsis* plants with the floral dip method. Homozygous lines were selected on media supplemented with kanamycin (50 mg/L). Seeds of AT5G58150::GUS lines were sown on B5 media with 1% sucrose (control) or additionally supplemented with 100 mM NaCl or 200 mM mannitol, left for stratification at 4°C for 2 days, and transferred to the growth chamber. After 8 days of growth, seedlings were collected and fixed in the cold, freshly prepared 90% (v/v) acetone solution for a minimum of 10 min and placed in the GUS staining solution containing 0.05% X-Gluc (Lab Empire) in 23.5 mM K3-phosphate buffer, pH 7.0, 1.25 mM potassium ferrocyanide, 1.25 mM potassium ferricyanide, 0.1% Triton X100. Plants were infiltrated in a desiccator connected to a vacuum pump (N820 Laboport, KNF) for 15 min, 130 mbar, and left at 37°C overnight in darkness. After that several rounds of washings were performed in an ethanol–water solution series (30%, 50%, 70%, 90%, 96%) until chlorophyll was removed. Samples were photographed using a Nikon camera or Zeiss Lumar V12 Fluorescence Stereomicroscope (KL 2500 LCD).

### Immunoprecipitation

35S::AT5G58150-GFP and 35S::GFP seeds were grown on a solid B5 medium with vitamins and 1% sucrose for 8 days. About 3 g of seedlings were collected and immersed into 75 ml ice-cold MC buffer (0.1 M sucrose, 10 mM sodium phosphate pH 7.0, 50 mM NaCl) with formaldehyde (final concentration 1%) added. Next vacuum infiltration was applied 3 times for 10 min. The fixation was stopped by adding 2.5 ml glycine (1.25 M stock) followed by vacuum application for 5 min. Then the seedlings were washed 3 times with the MC buffer. After buffer removal, the material was frozen in liquid nitrogen and stored at -80 °C. Seedlings were powdered in liquid nitrogen. All subsequent steps were carried out on the ice. 1 g of tissue was dissolved in 1 ml of immunoprecipitation lysis buffer containing 20 mM Tris HCl pH 7.5, 150 mM NaCl, 2 mM EDTA, 0.5% NP-40 (Thermo Fisher Scientific), 1 mM PMSF, 1 mM DTT, Complete Protease Inhibitor Cocktail (Roche). The suspension was sonicated (3 cycles of the 30 s with 30 s intervals at 40% of intensity) on a Bioruptor sonicator (Diagenode, USA). The material was incubated for 0.5 h in a cold room on a rotating platform, then centrifuged for 10 min at 4 °C at 5000 g. The supernatant was transferred to new tubes and centrifuged for 20 min at 4 °C at 20,000 g. The protein concentration of the supernatant was measured by the Bradford method. GFP-Trap_A beads (Chromotek) were used for protein immunoprecipitation. The resin was resuspended three times in 1 ml of dilution buffer containing 20 mM Tris HCl pH 7.5, 150 mM NaCl, 0.5 mM EDTA, and 1 mM DTT with cOmplete (Roche). To each sample containing 7 mg of protein in 7 ml of precipitation buffer, 7 ml of dilution buffer together with 25 µl of the resin was added. The samples were mixed for 1 h in the cold room. Then the supernatant was removed and washed with 1 ml of immunoprecipitation buffer mixed with dilution buffer in a ratio of 1:1. After incubation for 5 min, the resin was rinsed with 1 ml of dilution buffer without protease inhibitors for 5 min. The last step was repeated.

### Sample preparation for mass spectrometric (MS) analysis

To prepare the samples for the MS analysis, the protocol “On-bead digest protocol for mass spectrometry following immunoprecipitation with Nano-Traps”, provided by Chromotek was performed [77]. The GFP-Trap_A beads were resuspended in 500 μl of ice-cold lysis buffer and centrifuged at 2500 g for 2 min at 4 °C. The supernatant was discarded and the wash with the lysis buffer was repeated twice. Then the GFP-Trap_A beads were resuspended in 500 μl of ice-cold dilution buffer and centrifuged at 2500 g for 2 min at 4 °C. The supernatant was discarded and the wash with dilution buffer was repeated once. The GFP-Trap_A beads were resuspended in 25 μl of elution buffer I, containing 50 mM Tris–HCl pH 7.5, 2 M urea, 5 μg/ml Sequencing Grade Modified Trypsin (Promega), 1 mM DTT, incubated in a thermomixer (Thermo Fisher Scientific) at 30 °C at 400 rpm for 30 min, and centrifuged at 2500 g for 2 min at 4 °C. After transferring the supernatant to a fresh vial, the GFP-Trap_A beads were resuspended in 50 μl of elution buffer II, containing 50 mM Tris–HCl pH 7.5, 2 M urea, 5 mM iodoacetamide and centrifuged at 2500 g for 2 min at 4 °C. The supernatant was combined with the previous one and the procedure was repeated once. The samples were then incubated in the thermomixer at 32 °C at 400 rpm overnight in darkness. The reaction was stopped by adding 1 μl of trifluoroacetic acid. Peptides were washed two times with acetonitrile on paramagnetic beads (SpeedBead GE45152105050250 and GE65152105050250, Sigma-Aldrich, mixed in a ratio 1:1), and eluted in aqueous solution according to [78]. Samples were analysed in triplicates.

### Liquid chromatography and tandem mass spectrometry (LC–MS/MS)

Peptides were analysed using an UltiMate 3000 RSLCnano System coupled with a Q Exactive mass spectrometer (Thermo Fisher Scientific) through a DPV-550 Digital PicoView nanospray source (New Objective). The sample was loaded onto a trap column (Acclaim PepMap 100 C18, 75 μm × 20 mm, 3 μm particle, 100 Å pore size, Thermo Fisher Scientific) in 2% acetonitrile with 0.05% TFA at a flow rate of 5 μl/min and further resolved on an analytical column (Acclaim PepMap RSLC C18, 75 µm × 500 mm, 2 µm particle, 100 Å pore size, Thermo Fisher Scientific) with a 180 min gradient of acetonitrile (from 2 to 40%) in 0.05% formic acid at a flow rate of 250 nl/min. The Q Exactive was operated in a data-dependent mode using the Top12 method. Full-scan MS spectra were acquired with a resolution of 70,000 at m/z 200 with automatic gain control (AGC target) of 10^6^. The MS/MS spectra were acquired with a resolution of 17,500 at m/z 200 with an AGC target of 5·10^5^. The maximum ion accumulation times for the full MS and the MS/MS scans were 120 ms and 60 ms, respectively. Peptides were dynamically excluded from fragmentation within 30 s. The RAW file was processed by the Proteome Discoverer platform (v.1.4, Thermo Fisher Scientific) and searched using a locally installed MASCOT search engine (v.2.5.1, Matrix Science) against the SwissTrEMBL *Arabidopsis thaliana* database (release April 2022, 39,329 sequences). For peptide identification, a precursor ion mass tolerance of 10 ppm was used for the MS1 level and a fragment mass tolerance of 20 mmu was used for MS2. Only tryptic peptides with up to one missed cleavage site were considered. Oxidation of methionine and N-terminal protein acetylation were set as variable modifications, whereas carbamidomethylation on cysteines was set as a fixed modification. The maximum false discovery rate (FDR) of peptide identification was set to 1% using a decoy database strategy performed with the Percolator algorithm. Proteins were considered to be Y5815_ARATH interactors when they meet the following criteria: (i) proteins absent in CONTROL (35S::GFP) samples, but identified in at least 2 TEST 35S::At5g58150-GFP) samples (2/3), (ii) proteins identified with ≥ 2 peptides at least in one experiment in TEST samples, (iii) proteins identified with max average score ≥ 35 in TEST samples, and (iv) proteins identified in TEST samples with an average score at least 3 times higher than in CONTROL samples.

### Statistical analysis

Statistical analysis was performed using the R software [79]. *Gene expression*: The significance of the effects of the plant line and the light conditions on the log-transformed level of either the *PHOT2* or *AT5G58150* transcript was examined with two-way ANOVA with interaction. The pairwise comparisons of means were performed with Tukey’s method, using the *multcomp* [80] and *emmeans* packages. *Germination experiment*: The fraction of non-germinated seeds, as well as the fraction of seedlings at different stages of development, were treated as binomially-distributed variables. The relationship between response (*e.g.* the fraction of non-germinated seeds) and two predictors (plant line and concentration of either NaCl or mannitol), as well as the predictor interaction, was expressed as a generalized linear model, with the logit link function, using the *glm* package. The effects of the plant line, solute concentration, and their interaction were examined with the Type III Analysis of the Deviance procedure combined with the likelihood ratio χ^2^ test, using the *car* package. The differences in logarithms of odds ratios between particular groups were tested using *glht* command of the *multcomp* package, with Hommel’s adjustment used to control the familywise Type I error rate. The results of the analysis and sets of examined contrasts can be found in Supporting Tables S1 – S3. *Lateral root formation*: The influence of medium type (three levels: no solute added, 200 mM mannitol, 100 mM NaCl), plant line (three levels), and their interaction on either root length or lateral root density was expressed using a mixed linear model, with the experiment repetition (three levels) treated as a random factor. The model was fitted using the *lmer* command of the *lme4* package [81]. The lateral root count was assumed to follow Poisson's distribution [82] and a generalized mixed linear model was fitted using the *glmer* command. To assess the significance of the main effects and their interactions, the *car* package implementation of ANOVA (type III) was used, with Wald's χ^2^ test. Contrasts between means were examined using the *glht* command of the *multcomp* package with Hommel’s adjustment. The results of the statistical analysis of the root system properties are included in Supporting Tables S4 and S5. All *p-*values reported in the text and figures are adjusted for multiple comparisons.

### Supplementary Information

**Additional file 1: Fig. S1. **The locus of *PHOT2 (AT5G58140)* and *AT5G58140* genes in the whole-genome pairwise alignments between Arabidopsis and several plant genomes, obtained with the VISTA-Point tool (https://pipeline.lbl.gov/). For each species, the curves show the identity score of the alignment, averaged across a 100 bp moving window. A region is considered to be conserved if the sequence identity is at least 70% over at least 100 bp. Conserved regions are color-coded as dark blue, light blue or orange if they correspond to exons, UTRs, or non-coding sequences, respectively. **Fig. S2.** Confirmation of *at5g58150* mutation (*SALK_093781C*, insertion in the promoter region) with the three primers in one reaction (Lba1, sequence specific primers Table S1). **Fig. S3.** Kinase activity assay for AT5G58150 kinase domain. **Fig. S4.** Laser scanning confocal images of *N. benthamiana *epidermal cells transiently co-expressing AT5G58150-GFP or Plasma Membrane-mCherry or Tonoplast-mCherry markers. **Fig. S5.** Western Blot analysis of* N. benthamiana* epidermal cells transiently co-expressing PHOT2 and AT5G58150 fused with C(N)-terminal YFP fragments in the following configurations: AT5G58150_NtermYFP and AT5G58150_CtermYFP, NtermYFP_PHOT2 and PHOT2_CtermYFP, NtermYFP_PHOT2 and AT5G58150_CtermYFP, CtermYFP_PHOT2 and PHOT2_NtermYFP, PHOT2_CtermYFP and AT5G58150_NtermYFP, AT5G58150_NtermYFP, AT5G58150_CtermYFP, NtermYFP_PHOT2, CtermYFP_PHOT2, PHOT2_NtermYFP, PHOT2_CtermYFP probed with anti-cYFP and anti-nYFP. The white light image was merged with the chemiluminescent signal to show the borders of the membranes and molecular weight marker (PageRuler Prestained Protein, SM #26616, Thermo Scientific). Cropped image in Fig. 4. **Fig. S6.** AT5G58150 and phototropin2 interactions tested with MYTH assay. **Fig. S7.** Averaged curves (A) and amplitudes (B) of changes in rosette leaf transmittance T induced by blue light of increasing irradiance of 0.4, 1.6, 4, 20, 40, 80, 120 µmol·m^-2^·s^-1^ in wild type, *at5g58150 *mutant and 35S::AT5G58150-GFP lines. **Fig. S8.** Co-expression analysis of AT5G58150 based on proteomic data performed with Athena (https://athena.proteomics.wzw.tum.de/master_arabidopsisshiny/). Co-expression of AT5G58150 with BAK1, BSK, BRL1, BRL3 is observed. **Fig. S9.** Co-expression analysis of *AT5G58150* based on transcriptomic data performed with Athena (https://athena.proteomics.wzw.tum.de/master_arabidopsisshiny/). Co-expression of *AT5G58150* with *ATBS1, BES1, BZR1* is observed. **Table S6.** List of proteins that co-immunoprecipitated with AT5G59150-GFP, identified with Mass Spectrometry, which fulfill the interaction criteria, together with the values of parameters taken into account in the calculations. **Table S7.** List of proteins co-immunoprecipitated with AT5G59150-GFP, identified with MS, with their functional descriptions from the String database. **Table S8.** Primer sequences used in this study. **Table S9.** Genes co-expressed with *AT5G58150* based on published data from transcriptomic analysis performed with the Arabidopsis Co-expression Tool (https://www.michalopoulos.net/act). Genes of the brassinosteroid pathway, involved in root development as well as cell wall and junction formation, are marked in yellow.

**Additional file 2: Table S1. **Results of the statistical analysis of the differences between wild type plants and *at5g58150*/ AT5G58150-GFP lines in fraction of non-germinated seeds two (A, C) or three (B, D) days after end of stratification on media supplemented with different concentrations of NaCl (A, B) or mannitol (C, D). A logistic regression model was fitted using the *glm *command of the R software. Type III analysis of deviance was performed using the *Anova* command of the *car* package, with the effects coding of factor levels. Differences between natural logarithms of odds ratios were tested with the *glht*command of the *multcomp* package, with a custom contrast matrix. *p* values are adjusted for multiple comparisons using Hommel’s method. **Table S2.** Results of the statistical analysis of the differences between wild type plants and at5g58150/ AT5G58150-GFP lines in fraction of seeds that germinated (radicles emerged), but the seedlings did not open cotyledons. **Table S3.** Results of the statistical analysis of the differences in mean fraction of seeds that germinated and opened cotyledons, two (A, C) or three (B, D) days after end of stratification on media supplemented with different concentrations of NaCl (A, B) or mannitol (C, D). **Table S4.** Results of the statistical analysis of the differences in mean root length (A), the number of lateral roots per cm of the primary root (B) and lateral root count (C) of 9-day-old *Arabidopsis* seedlings. **Table S5.** Results of the statistical analysis of the interaction contrasts (differences of differences) for lateral root density (the number of lateral roots per cm of the primary root) of 9-day-old *Arabidopsis* seedlings.

## Data Availability

Data analysed during this study are included in this published article and its supplementary information files. The datasets used and/or analysed during the current study are available from the corresponding author upon reasonable request. The mass spectrometry data were deposited to the ProteomeXchange Consortium [83] via the MassIVE repository with the dataset identifier PXD039040. The 3’RACE datasets generated during the current study are available in the GenBank repository (https://www.ncbi.nlm.nih.gov/genbank/), *AT5G58150*—OQ674723 (https://www.ncbi.nlm.nih.gov/nuccore/OQ674723) and* PHOT2*—OQ674724 (https://www.ncbi.nlm.nih.gov/nuccore/OQ674724).
