# Supplementary material for: Phototropin2 3’UTR overlaps with the AT5G58150 gene encoding an inactive RLK kinase
Source: BMC Plant Biol. 2024 Jan 18;24:55. doi: 10.1186/s12870-024-04732-2 (PMC10795372; doi:10.1186/s12870-024-04732-2)
Supplement: Supplementary file 1 — Additional file 1: Fig. S1. The locus of PHOT2 (AT5G58140) and AT5G58140 genes in the whole-genome pairwise alignments between Arabidopsis and several plant genomes, obtained with the VISTA-Point tool (https://pipeline.lbl.gov/). For each species, the curves show the identity score of the alignment, averaged across a 100 bp moving window. A region is considered to be conserved if the sequence identity is at least 70% over at least 100 bp. Conserved regions are color-coded as dark blue, light blue or orange if they correspond to exons, UTRs, or non-coding sequences, respectively. Fig. S2. Confirmation of at5g58150 mutation (SALK_093781C, insertion in the promoter region) with the three primers in one reaction (Lba1, sequence specific primers Table S1). Fig. S3. Kinase activity assay for AT5G58150 kinase domain. Fig. S4. Laser scanning confocal images of N. benthamiana epidermal cells transiently co-expressing AT5G58150-GFP or Plasma Membrane-mCherry or Tonoplast-mCherry markers. Fig. S5. Western Blot analysis of N. benthamiana epidermal cells transiently co-expressing PHOT2 and AT5G58150 fused with C(N)-terminal YFP fragments in the following configurations: AT5G58150_NtermYFP and AT5G58150_CtermYFP, NtermYFP_PHOT2 and PHOT2_CtermYFP, NtermYFP_PHOT2 and AT5G58150_CtermYFP, CtermYFP_PHOT2 and PHOT2_NtermYFP, PHOT2_CtermYFP and AT5G58150_NtermYFP, AT5G58150_NtermYFP, AT5G58150_CtermYFP, NtermYFP_PHOT2, CtermYFP_PHOT2, PHOT2_NtermYFP, PHOT2_CtermYFP probed with anti-cYFP and anti-nYFP. The white light image was merged with the chemiluminescent signal to show the borders of the membranes and molecular weight marker (PageRuler Prestained Protein, SM #26616, Thermo Scientific). Cropped image in Fig. 4. Fig. S6. AT5G58150 and phototropin2 interactions tested with MYTH assay. Fig. S7. Averaged curves (A) and amplitudes (B) of changes in rosette leaf transmittance T induced by blue light of increasing irradiance of 0.4, 1.6, 4, 20, 40, 80, 120 µmol·m-2·s-1 in wild type, at5g5 [file 12870_2024_4732_MOESM1_ESM.docx]

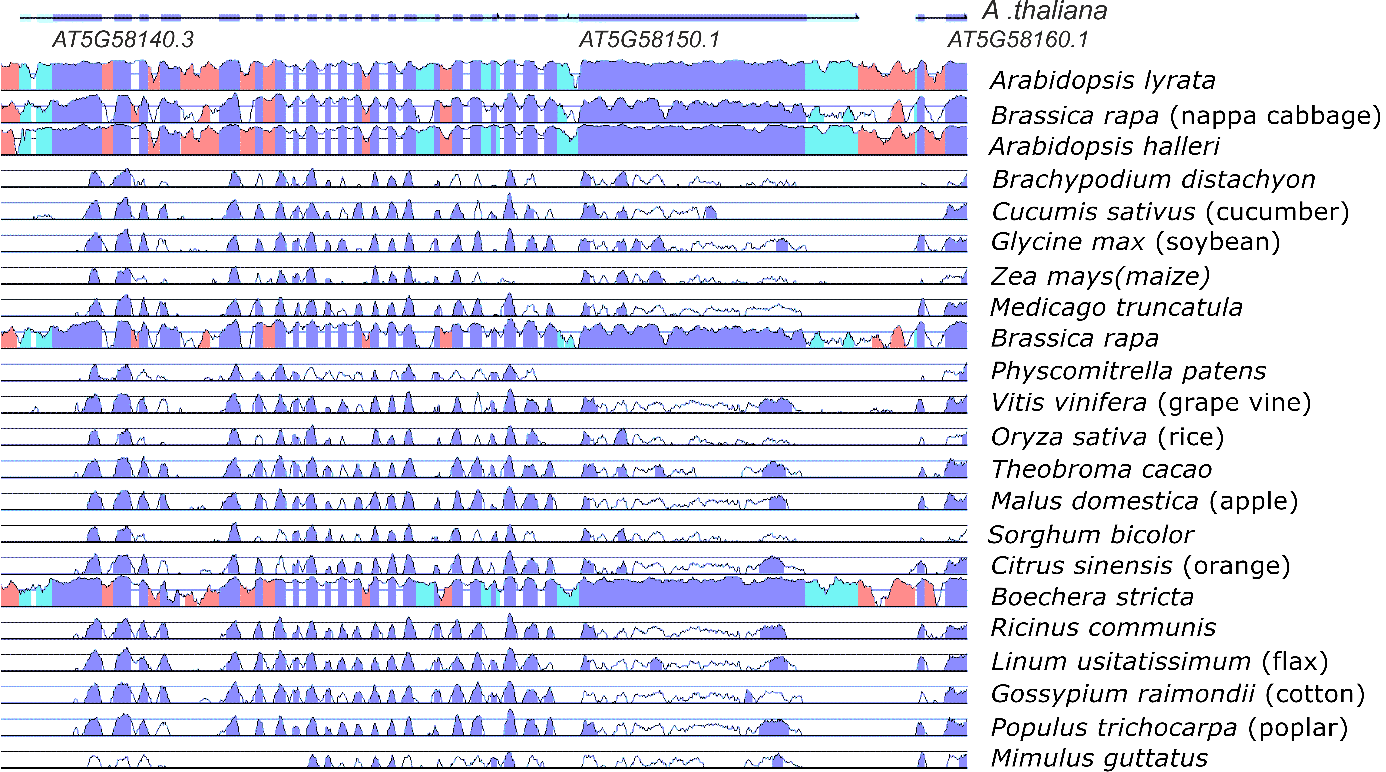


Fig. S1. The locus of *PHOT2* (*AT5G58140*) and *AT5G58140* genes in the whole-genome pairwise alignments between *Arabidopsis* and several plant genomes, obtained with the VISTA-Point tool (<https://pipeline.lbl.gov/>). For each species, the curves show the identity score of the alignment, averaged across a 100 bp moving window. A region is considered to be conserved if the sequence identity is at least 70% over at least 100 bp. Conserved regions are color-coded as dark blue, light blue or orange if they correspond to exons, UTRs, or non-coding sequences, respectively.


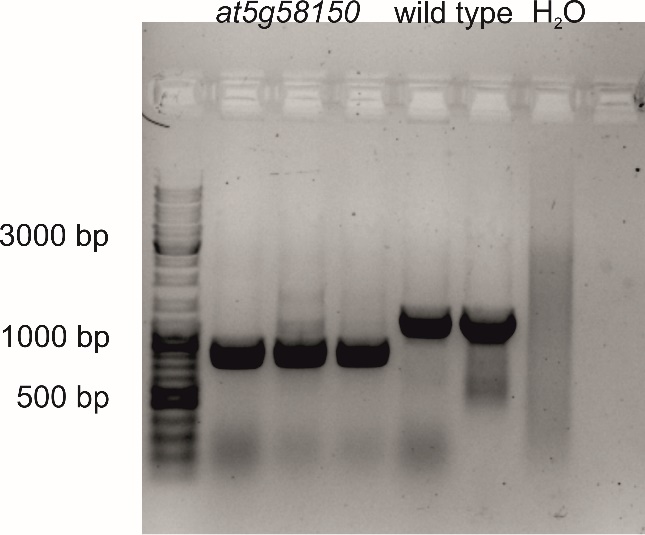


Fig. S2. Confirmation of *at5g58150* mutation (*SALK_093781C*, insertion in the promoter region) with the three primers in one reaction (Lba1, sequence specific primers Table S1). The predicted product size for WT: 1183bp, the product size for the *at5g58150* mutation: 506-806 bp (calculated by T-DNA Primer Design tool: http://signal.salk.edu/tdnaprimers.2.html). DNA was separated in 1% agarose in TAE buffer and stained with Midori Green.


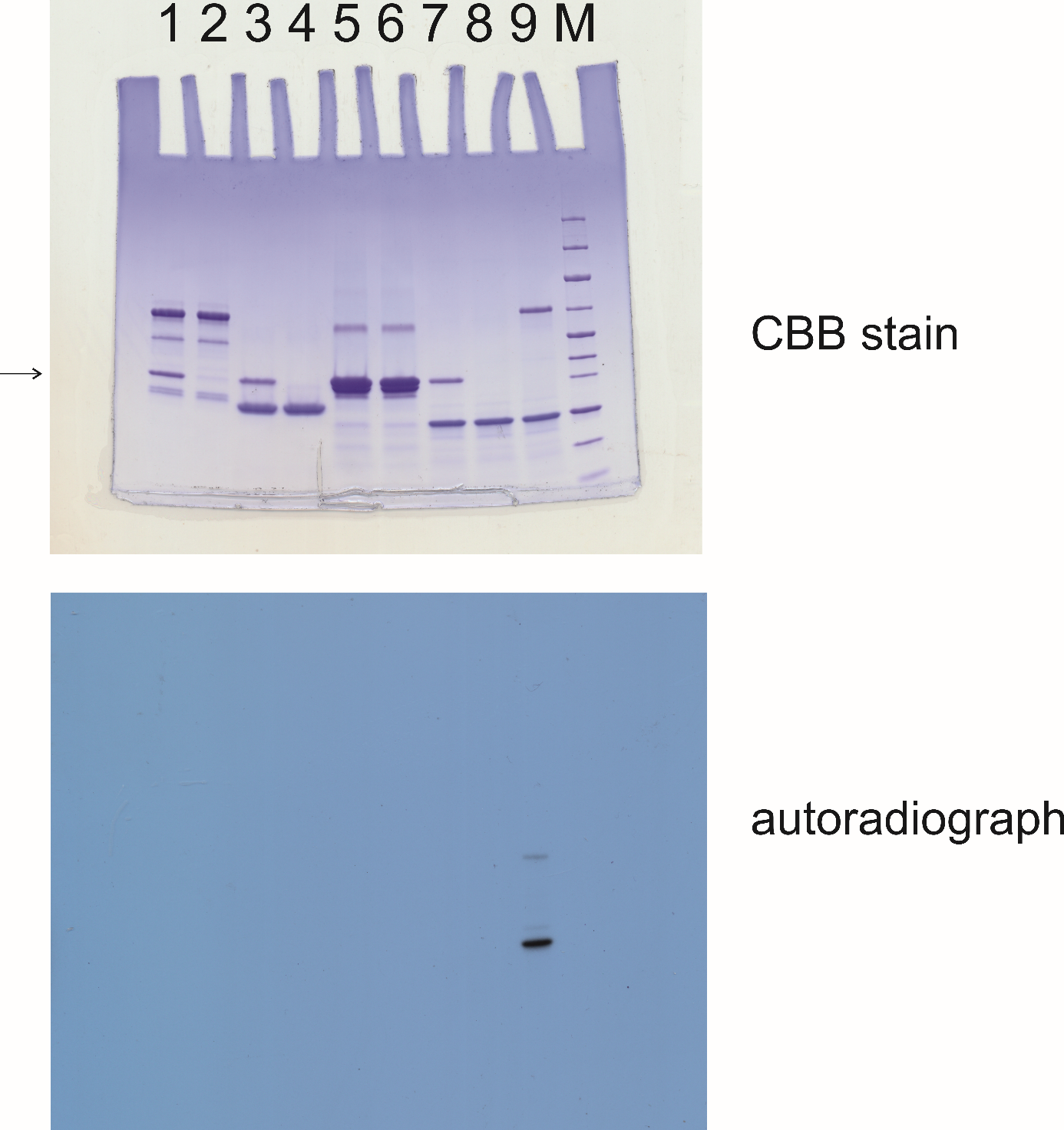


Fig. S3. Kinase activity assay for AT5G58150 kinase domain. 2 μg of recombinant purified kinase domain was incubated with 10 μg of putative substrate and with 50 μM ATP supplemented with 1 μCi of [γ-^32^P]ATP in the kinase buffer (25 mM Tris-HCl, pH 7.5, 5 mM EGTA, 1 mM DTT, 30 mM MgCl_2_, total reaction volume 25 μl) at 30°C for 30 min. Kinase activities were tested using GST-THRUMIN1 (lines 1 and 2), histone (lines 3 and 4), casein (lines 5 and 6), and MBP (Myelin Basic Protein, lines 7, 8, 9). Odd lines contain the kinase, even lines do not, and serve as the control. The arrow indicates the position of the purified AT5G58150 kinase domain. As a control for kinase activity, GST-SnRK2.6 was used (line 9), showing both substrate phosphorylation and autophosphorylation. marker: 26614 PageRuler™ Unstained Protein Ladder Thermo Fisher Scientific.


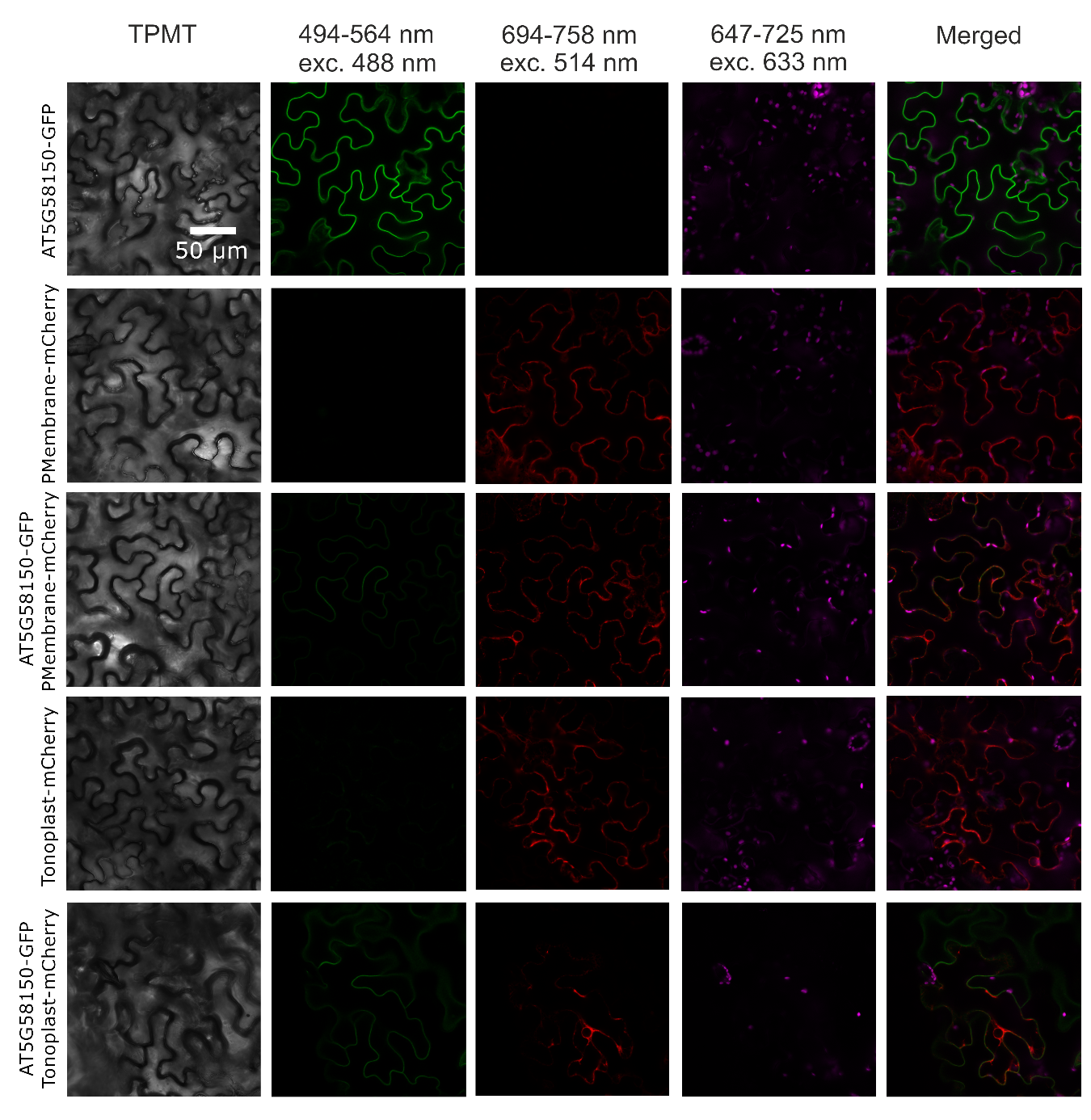


Fig. S4. Laser scanning confocal images of *N. benthamiana* epidermal cells transiently co-expressing AT5G58150-GFP or Plasma Membrane-mCherry or Tonoplast-mCherry markers. mCherry fluorescence is in red, and GFP fluorescence is in green, chlorophyll fluorescence in magenta.


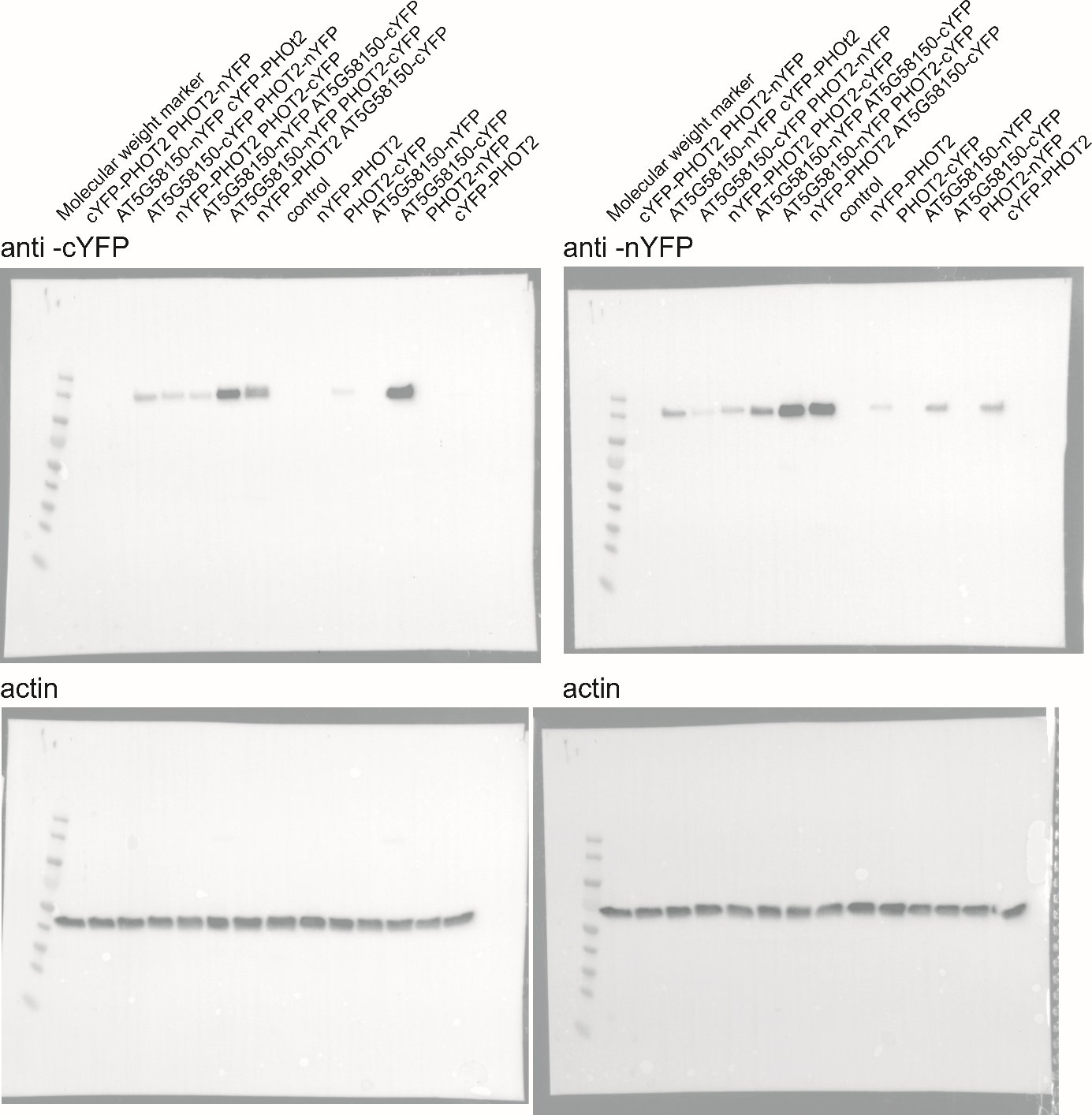


Fig. S5. Western Blot analysis of *N. benthamiana* epidermal cells transiently co-expressing PHOT2 and AT5G58150 fused with C(N)-terminal YFP fragments in the following configurations: AT5G58150__Nterm_YFP and AT5G58150__Cterm_YFP, _Nterm_YFP_PHOT2 and PHOT2__Cterm_YFP, _Nterm_YFP_PHOT2 and AT5G58150__Cterm_YFP, _Cterm_YFP_PHOT2 and PHOT2__Nterm_YFP, PHOT2__Cterm_YFP and AT5G58150__Nterm_YFP, AT5G58150__Nterm_YFP, AT5G58150__Cterm_YFP, _Nterm_YFP_PHOT2, _Cterm_YFP_PHOT2, PHOT2__Nterm_YFP, PHOT2__Cterm_YFP probed with anti-cYFP and anti-nYFP. The white light image was merged with the chemiluminescent signal to show the borders of the membranes and molecular weight marker (PageRuler Prestained Protein, SM #26616, Thermo Scientific). Cropped image in Fig. 4.


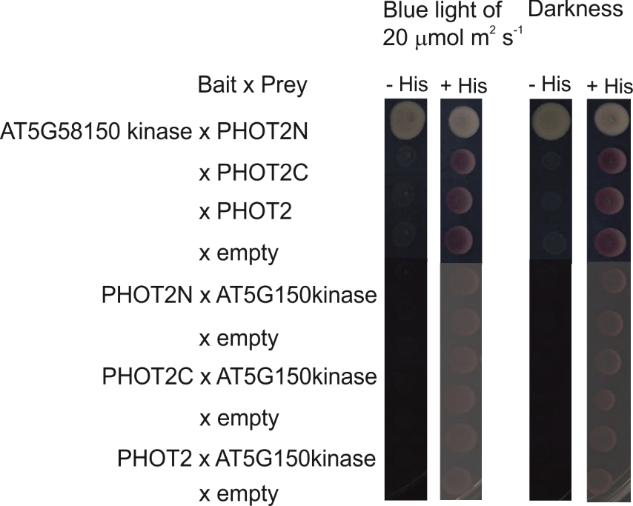


Fig. S6. AT5G58150 and phototropin2 interactions tested with MYTH assay. The AT5G58150 kinase domain, full-length PHOT2 and its N/C-terminal parts were used as baits and preys. Overnight cultures of transformed yeasts were plated on the solid SC-Leu-Trp (+His) medium serving as a control, SC-Leu-Trp-His (-His) solid selection medium supplemented with 5 mM 3-aminotriazole (3-AT). Yeast plated on solid media were cultured in darkness or under blue light (~20 μmol·m^−2^·s^−1^, 470 nm) in 30°C for 3 days. For all bait/prey constructs, a co-transformation with empty prey/bait vectors was performed to avoid false-positive signals being a result of a non-specific self-activation. The results represent one of three independent biological replicates.


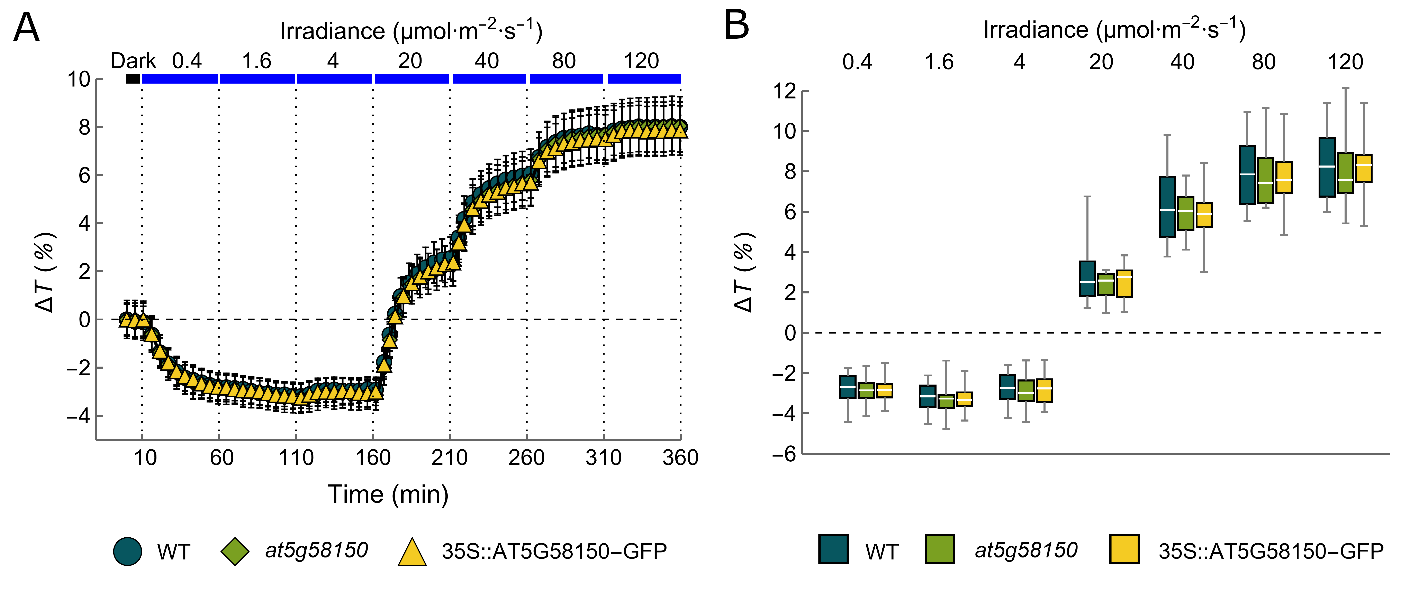


Fig. S7. Averaged curves (A) and amplitudes (B) of changes in rosette leaf transmittance *T* induced by blue light of increasing irradiance of 0.4, 1.6, 4, 20, 40, 80, 120 µmol·m^-2^·s^-1^ in wild type, *at5g58150* mutant and 35S::AT5G58150-GFP lines. Blue bars above the plot in A marks subsequent phases of irradiation. Error bars in A show SE. Boxes in B mark the interquartile range, whiskers show data range, horizontal bars across the boxes marks the median. For each plant line, 15 – 18 biological replicates (leaves from different plants) were examined. Plants were dark-adapted for at least 16 h before the measurement.


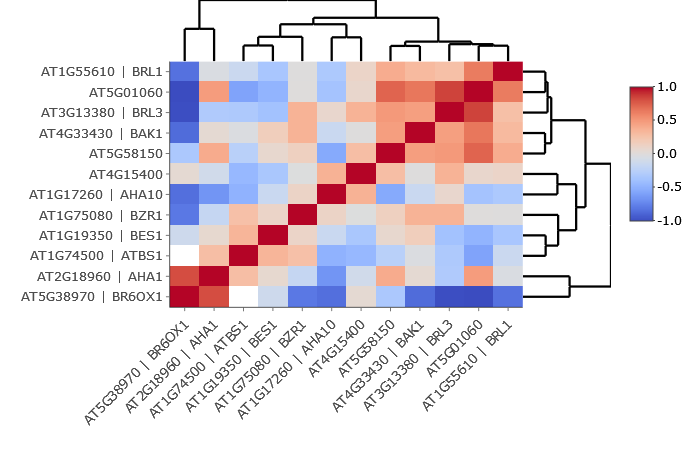


Fig. S8. Co-expression analysis of AT5G58150 based on proteomic data performed with Athena (https://athena.proteomics.wzw.tum.de/master_arabidopsisshiny/). Co-expression of AT5G58150 with BAK1, BSK, BRL1, BRL3 is observed.


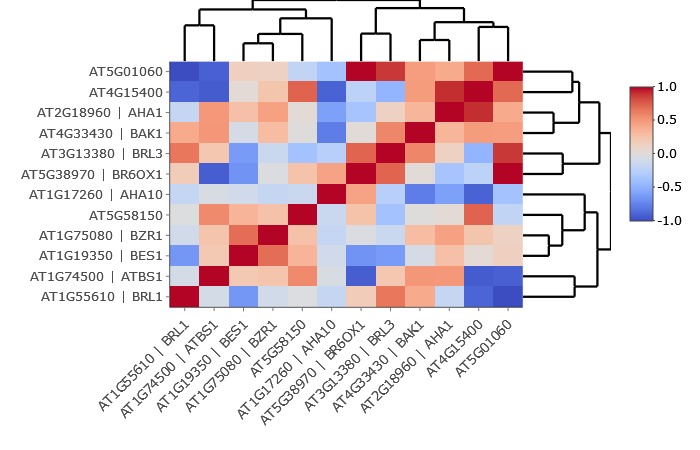


Fig. S9. Co-expression analysis of AT5G58150 based on transcriptomic data performed with Athena (https://athena.proteomics.wzw.tum.de/master_arabidopsisshiny/). Co-expression of AT5G58150 with *ATBS1, BES1, BZR1* is observed.

Table S6. List of proteins that co-immunoprecipitated with AT5G59150-GFP, identified with Mass Spectrometry, which fulfill the interaction criteria, together with the values of parameters taken into account in the calculations.

| Protein name | Number of valid values in TEST samples | Protein Max score In TEST samples | Σ# Peptides | Σ Protein Sequence Coverage [%] | TEST Average Score/ CONTROL Average Score |
| --- | --- | --- | --- | --- | --- |
| AT5G58150 | 3 | 2961.70 | 26 | 40.76 | Not detected in CONTROL |
| LHB1B2 | 3 | 399.80 | 4 | 11.32 | Not detected in CONTROL |
| PSBO2 | 2 | 451.16 | 13 | 42.30 | Not detected in CONTROL |
| At1g07930 | 3 | 222.80 | 4 | 13.71 | Not detected in CONTROL |
| HA1 | 3 | 157.27 | 15 | 19.39 | Not detected in CONTROL |
| CPN60B | 3 | 156.20 | 8 | 17.00 | Not detected in CONTROL |
| RBCS2B | 3 | 155.39 | 6 | 44.20 | Not detected in CONTROL |
| AT1G51805 | 3 | 123.05 | 7 | 7.35 | Not detected in CONTROL |
| AT2G37190 | 3 | 92.53 | 2 | 20.48 | Not detected in CONTROL |
| CAT | 3 | 112.81 | 3 | 6.96 | Not detected in CONTROL |
| PATL2 | 2 | 149.14 | 10 | 22.55 | Not detected in CONTROL |
| AT1G53430 | 2 | 105.12 | 3 | 3.21 | Not detected in CONTROL |
| LOS2 | 3 | 69.89 | 6 | 24.71 | Not detected in CONTROL |
| CYSC1 | 3 | 82.59 | 3 | 8.97 | Not detected in CONTROL |
| RPL5A | 2 | 115.91 | 6 | 21.05 | Not detected in CONTROL |
| HA3 | 3 | 73.48 | 7 | 10.12 | Not detected in CONTROL |
| AT3G11630 | 3 | 64.91 | 3 | 16.92 | Not detected in CONTROL |
| F5M15.5 | 2 | 114.59 | 5 | 14.52 | Not detected in CONTROL |
| AT2G27720 | 2 | 80.89 | 3 | 49.45 | Not detected in CONTROL |
| RPL24 | 3 | 59.37 | 2 | 11.11 | Not detected in CONTROL |
| AT3G17410 | 3 | 65.79 | 2 | 6.32 | Not detected in CONTROL |
| AT3G47370 | 2 | 75.58 | 3 | 25.41 | Not detected in CONTROL |
| GRF10 | 2 | 101.83 | 4 | 17.93 | Not detected in CONTROL |
| HIR2 | 2 | 91.82 | 3 | 8.07 | Not detected in CONTROL |
| RABA1d | 2 | 83.53 | 2 | 9.81 | Not detected in CONTROL |
| AT1G11330 | 3 | 67.80 | 2 | 2.81 | Not detected in CONTROL |
| UNE5 | 2 | 78.64 | 2 | 8.65 | Not detected in CONTROL |
| J3 | 3 | 44.74 | 2 | 5.83 | Not detected in CONTROL |
| NSP3 | 2 | 63.04 | 2 | 4.93 | Not detected in CONTROL |
| ATP3 | 2 | 59.02 | 2 | 7.69 | Not detected in CONTROL |
| AT4G14060 | 2 | 58.10 | 2 | 11.92 | Not detected in CONTROL |
| EMB3137 | 2 | 46.48 | 2 | 16.41 | Not detected in CONTROL |
| AT3G09630 | 2 | 62.69 | 5 | 15.56 | Not detected in CONTROL |
| ADK2 | 2 | 56.94 | 2 | 10.27 | Not detected in CONTROL |
| BSK8 | 2 | 47.12 | 3 | 8.83 | Not detected in CONTROL |
| CRK11 | 2 | 43.28 | 3 | 7.98 | Not detected in CONTROL |
| CPK33 | 2 | 42.65 | 2 | 6.71 | Not detected in CONTROL |
| GOX1 | 2 | 47.84 | 2 | 6.27 | Not detected in CONTROL |
| LRK1 | 2 | 37.66 | 2 | 3.11 | Not detected in CONTROL |
| AT4G08850 | 3 | 195.81 | 7 | 8.61 | 15.32 |
| CNX1 | 3 | 103.87 | 7 | 16.04 | 6.33 |
| HA2 | 3 | 138.31 | 14 | 17.72 | 6.06 |
| AT3G61260 | 3 | 115.81 | 3 | 13.68 | 6.05 |
| ACA10 | 3 | 82.43 | 2 | 2.71 | 5.93 |
| RPS7.1 | 2 | 51.88 | 2 | 18.06 | 4.25 |
| AT5G47190 | 2 | 85.32 | 3 | 13.10 | 4.16 |
| PSBO1 | 2 | 531.95 | 13 | 40.96 | 3.43 |
| EMB2207 | 2 | 73.56 | 5 | 18.45 | 3.42 |
| BSK1 | 3 | 70.23 | 3 | 8.40 | 3.34 |

Table S7. List of proteins co-immunoprecipitated with AT5G59150-GFP, identified with MS, with their functional descriptions from the String database.

| Protein name | Description |
| --- | --- |
| AT5G58150 | Leucine-rich repeat protein kinase family protein; Its function is described as protein serine/threonine kinase activity, Located in plasma membrane, vacuole; Expressed in 9 plant structures; Expressed during F mature embryo stage, petal differentiation and expansion stage, E expanded cotyledon stage, D bilateral stage |
| LHB1B2 | Light-harvesting complex ii chlorophyll a/b binding protein 1; Photosystem II light harvesting complex gene B1B2; The light-harvesting complex (LHC) functions as a light receptor, it captures and delivers excitation energy to photosystems with which it is closely associated |
| PSBO2 | Oxygen-evolving enhancer protein 1-2, chloroplastic; Encodes a protein which is an extrinsic subunit of photosystem II and which has been proposed to play a central role in stabilization of the catalytic manganese cluster. PsbO2 is the minor isoform in the wild-type. Mutants defective in this gene have been shown to be affected in the dephosphorylation of the D1 protein of PSII |
| AT1G07920 | GTP binding Elongation factor Tu family protein; Its function is described as calmodulin binding, translation elongation factor activity; Involved in translational elongation; Located in mitochondrion, vacuole; Expressed in cotyledon, male gametophyte, guard cell, juvenile leaf, leaf; Expressed during seedling growth |
| HA1 | ATPase 1, plasma membrane-type; The plasma membrane H(+) ATPase of plants and fungi generates a proton gradient that drives the active transport of nutrients by H(+)-symport. The resulting external acidification and/or internal alkinization may mediate growth responses. Forms a functional cation-translocating unit with CNGC17 that is activated by PSKR1/BAK1 and other BAK1/RLK complexes |
| CPN60B | Chaperonin 60 subunit beta 1, chloroplastic; Encodes the beta subunit of the chloroplast chaperonin 60, a homologue of bacterial GroEL. Mutants in this gene develops lesions on its leaves, expresses systemic acquired resistance (SAR) and develops accelerated cell death to heat shock stress. |
| RBCS2B | Ribulose bisphosphate carboxylase small chain 2B, chloroplastic; RuBisCO catalyzes two reactions: the carboxylation of D- ribulose 1,5-bisphosphate, the primary event in carbon dioxide fixation, as well as the oxidative fragmentation of the pentose substrate. Both reactions occur simultaneously and in competition at the same active site; Belongs to the RuBisCO small chain family |
| AT1G51805 | Leucine-rich repeat protein kinase family protein; Its function is described as protein serine/threonine kinase activity; Located in endomembrane system; Expressed in 20 plant structures; Expressed during 13 growth stages |
| AT2G37190 | Large subunit ribosomal protein l12e; Ribosomal protein L11 family protein; Binds directly to 26S ribosomal RNA |
| CAT | Catalase-2; Encodes a peroxisomal catalase, highly expressed in bolts and leaves. mRNA expression patterns show circadian regulation with mRNA levels being high in the subjective early morning. Loss of function mutations have increased H_2_O_2_ levels and increased H_2_O_2_ sensitivity. Mutants accumulate more toxic ions yet show decreased sensitivity to Li^+^. This decreased sensitivity is most likely due to an insensitivity to ethylene. |
| PATL2 | PATELLIN 2; Carrier protein that may be involved in membrane- trafficking events associated with cell plate formation during cytokinesis. Binds to some hydrophobic molecules such as phosphoinositides and promotes their transfer between the different cellular sites; Belongs to the patellin family |
| AT1G53430 | Probable LRR receptor-like serine/threonine-protein kinase Leucine-rich repeat transmembrane protein kinase; Its function is described as kinase activity; Involved in protein amino acid phosphorylation; Located in plasma membrane; Expressed in 23 plant structures; Expressed during 13 growth stages |
| LOS2 | Bifunctional enolase 2/transcriptional activator; Multifunctional enzyme that acts as an enolase involved in the metabolism and as a positive regulator of cold-responsive gene transcription. Binds to the cis-element the gene promoter of STZ/ZAT10, a zinc finger transcriptional repressor |
| CYSC1 | Bifunctional L-3-cyanoalanine synthase/cysteine synthase C1, mitochondrial; Acts as a major beta-cyanoalanine synthase. The cyanoalanine synthesis reaction is more efficient than the cysteine synthase activity. Probably unable to interact with SAT and to form the decameric Cys synthase complex (CSC) and is therefore not an enzymatically true OASTL protein. Probably involved in the detoxification of cyanide that arises from ethylene biosynthesis. Maintains a low level of cyanide for proper root hair development |
| PL5A | 60S ribosomal protein L5-1; Component of the ribosome, a large ribonucleoprotein complex responsible for the synthesis of proteins in the cell. The small ribosomal subunit (SSU) binds messenger RNAs (mRNAs) and translates the encoded message by selecting cognate aminoacyl- transfer RNA (tRNA) molecules. The large subunit (LSU) contains the ribosomal catalytic site termed the peptidyl transferase center (PTC), which catalyzes the formation of peptide bonds, thereby polymerizing the amino acids delivered by tRNAs into a polypeptide chain |
| HA3 | Arabidopsis h(+)-atpase; ATPase 3, plasma membrane-type; The plasma membrane H(+) ATPase of plants and fungi generates a proton gradient that drives the active transport of nutrients by H(+)-symport. The resulting external acidification and/or internal alkinization may mediate growth responses; Belongs to the cation transport ATPase (P-type) (TC 3.A.3) family. Type IIIA subfamily |
| AT3G11630 | 2-Cys peroxiredoxin BAS1, chloroplastic; Thiol-specific peroxidase that catalyzes the reduction of hydrogen peroxide and organic hydroperoxides to water and alcohols, respectively. Plays a role in cell protection against oxidative stress by detoxifying peroxides. May be an antioxidant enzyme in the developing shoot and photosynthesizing leaf; Belongs to the peroxiredoxin family. AhpC/Prx1 subfamily |
| F5M15.5 | Senescence 2; Catalase-3; Occurs in almost all aerobically respiring organisms and serves to protect cells from the toxic effects of hydrogen peroxide |
| AT2G27720 | 60S acidic ribosomal protein family; Its function is described as structural constituent of ribosome; Involved in translational elongation, response to cold; Located in cytosol, cytosolic ribosome, ribosome, nucleus |
| RPL24 | Translation protein SH3-like family protein; One of two assembly initiator proteins, it binds directly to the 5'-end of the 23S rRNA, where it nucleates assembly of the 50S subunit. Required for optimal plastid performance in terms of photosynthesis and growth. Required for the translation of plastid mRNAs. Plays a critical role in biosynthesis of thylakoid membrane proteins encoded by chloroplast genes |
| AT3G17410 | Protein kinase superfamily protein; Its function is described as protein serine/threonine kinase activity; Involved in protein amino acid phosphorylation, N-terminal protein myristoylation; Located in plasma membrane; |
| AT3G47370 | Ribosomal protein S10p/S20e family protein; Its function is described as structural constituent of ribosome, RNA binding; Involved in translation; Located in 6 components; Expressed in 23 plant structures |
| GRF10 | 14-3-3 PROTEIN G-BOX FACTOR14 EPSILON; 14-3-3-like protein GF14 epsilon; Encodes a 14-3-3 protein. This protein is reported to interact with the BZR1 transcription factor involved in brassinosteroid signaling and may affect the nucleocytoplasmic shuttling of BZR1 |
| HIR2 | SPFH/Band 7/PHB domain-containing membrane-associated protein family; Involved in N-terminal protein myristoylation; Located in mitochondrion, plasma membrane, vacuole, membrane; Expressed in 21 plant structures; Expressed during 9 growth stages; Contains the following InterPro domains: Band 7 protein (InterPro:IPR001107); BEST Arabidopsis thaliana protein match is: SPFH/Band 7/PHB domain-containing membrane-associated protein family |
| RABA1d | Ras-related protein RABA1d; Intracellular vesicle trafficking and protein transport |
| AT1G11330 | G-type lectin S-receptor-like serine/threonine-protein kinase: S-locus lectin protein kinase family protein; Its function is described as in 6 functions; Involved in protein amino acid phosphorylation, recognition of pollen; Located in plasma membrane; Expressed in 21 plant structures; Expressed during 13 growth stages |
| UNE5 | protein disulfide isomerase 11; Encodes a disulfide isomerase-like (PDIL) protein, a member of a multigene family within the thioredoxin (TRX) superfamily. Transcript levels for this gene are up-regulated in response to three chemical inducers of ER stress (dithiothreitol, beta-mercaptoethanol, and tunicamycin) |
| J3 | Chaperone protein dnaJ 3; Have a role in plant development probably in the structural organization of compartments; Belongs to the DnaJ family. A/I subfamily |
| NSP3 | Encodes a nitrile-specifier protein NSP3. NSP3 is one out of five (At3g16400/NSP1, At2g33070/NSP2, At3g16390/NSP3, At3g16410/NSP4 and At5g48180/NSP5) A. thaliana epithiospecifier protein (ESP) homologues that promote simple nitrile, but not epithionitrile or thiocyanate formation |
| ATP3 | ATP synthase subunit gamma, mitochondrial; Mitochondrial membrane ATP synthase (F(1)F(0) ATP synthase or Complex V) produces ATP from ADP in the presence of a proton gradient across the membrane which is generated by electron transport complexes of the respiratory chain |
| AT4G14060 | Polyketide cyclase/dehydrase and lipid transport superfamily protein; Involved in response to biotic stimulus, defense response; Expressed in cotyledon, root; |
| EMB3137 | 30S ribosomal protein S13, chloroplastic; Located at the top of the head of the 30S subunit, it contacts several helices of the 16S rRNA; Belongs to the universal ribosomal protein uS13 family |
| AT3G09630 | Ribosomal protein L4/L1 family; Its function is described as structural constituent of ribosome; Involved in translation |
| ADK2 | Encodes adenosine kinase 2, a typical, constitutively expressed housekeeping enzyme. Shows a high sequence identity with ADK1. Involved in salvage synthesis of adenylates and methyl recycling. Enzyme activity is substantially inhibited in roots, siliques and dry seeds by an unknown compound. May contribute to cytokinin interconversion; Belongs to the carbohydrate kinase PfkB family |
| BSK8 | Protein kinase protein with tetratricopeptide repeat domain; Its function is described as binding, protein kinase activity; Involved in protein amino acid phosphorylation, N-terminal protein myristoylation; Located in plasma membrane; Expressed in 22 plant structures; Expressed during 13 growth stages |
| CRK11 | Cysteine-rich RLK (RECEPTOR-like protein kinase) 11; Encodes putative receptor-like protein kinase that is induced by the soil-borne vascular bacteria, Ralstonia solanacearum. |
| CPK33 | Calcium-dependent protein kinase 33; Ca(^2+^)-dependent protein kinase. Negative regulator of stomatal closure and slow anion currents. Unable to phosphorylate THI1 in vitro, but the kinase activity is essential for the stomatal closure regulation. Phosphorylates FD. May play a role in signal transduction pathways that involve calcium as a second messenger |
| GOX1 | Aldolase-type TIM barrel family protein; Its function is described as glycolate oxidase activity; Involved in oxidation reduction, metabolic process; Located in 7 components; Expressed in cotyledon, fruit, guard cell, juvenile leaf, leaf; Expressed during seedling growth |
| LRK1 | L-type lectin-domain containing receptor kinase IV.1; Induced in response to Salicylic acid; In the C-terminal section; belongs to the protein kinase superfamily. Ser/Thr protein kinase family |
| AT4G08850 | Leucine-rich repeat receptor-like protein kinase family protein; Involved in the pollen tube perception of the female signal |
| CNX1 | Calnexin homolog 1; Calcium-binding protein that interacts with newly synthesized glycoproteins in the endoplasmic reticulum. It may act in assisting protein assembly and/or in the retention within the ER of unassembled protein subunits. It seems to play a major role in the quality control apparatus of the ER by the retention of incorrectly folded proteins |
| HA2 | ATPase 2, plasma membrane-type; The plasma membrane H(+) ATPase of plants and fungi generates a proton gradient that drives the active transport of nutrients by H(+)-symport. The resulting external acidification and/or internal alkinization may mediate growth responses. Involved in maintaining the membrane potential and delta-pH, together forming the plasma membrane protonmotive force (PMF) required for root and hypocotyl elongation and root tropism. Important for root growth and development during different nitrogen regimes. Forms a functional cation- translocating unit with CNGC17 th |
| AT3G61260 | Remorin family protein; Its function is described as binding; Located in plasma membrane, vacuole; Expressed in 24 plant structures; during 16 growth stages |
| ACA10 | Calcium-transporting ATPase 10, plasma membrane-type; This magnesium-dependent enzyme catalyzes the hydrolysis of ATP coupled with the translocation of calcium from the cytosol into the endoplasmic reticulum |
| RPS7.1 | 30S ribosomal protein S7, chloroplastic; Ribosomal protein S7p/S5e family protein; One of the primary rRNA binding proteins, it binds directly to 16S rRNA where it nucleates assembly of the head domain of the 30S subunit |
| AT5G47190 | 50S ribosomal protein L19-2, chloroplastic; Located at the 30S-50S ribosomal subunit interface and binds directly to 23S ribosomal RNA |
| PSBO1 | Oxygen-evolving enhancer protein 1-1, chloroplastic; Encodes a protein which is an extrinsic subunit of photosystem II and which has been proposed to play a central role in stabilization of the catalytic manganese cluster. In Arabidopsis thaliana the PsbO proteins are encoded by two genes: psbO1 and psbO2 . PsbO1 is the major isoform in the wild-type |
| EMB2207 | Large subunit ribosomal protein l3e; Encodes a cytoplasmic ribosomal protein; Belongs to the universal ribosomal protein uL3 family |
| BSK1 | Probable serine/threonine-protein kinase; Encodes BR-signaling kinase 1 (BSK1), one of the three homologous BR-signaling kinases (BSK1, AT4G35230; BSK2, AT5G46570; BSK3, AT4G00710). Mediates signal transduction from receptor kinase BRI1 by functioning as the substrate of BRI1. Plasma membrane localized |

Table S8. Primer sequences used in this study.

| Name | 5’-3’ Primer sequence |
| --- | --- |
| pORER1_AT5G58150_HidIII_for | GATAAG CTTTCT TGATTT GTAAAA CTCC |
| pORER1_AT5G58150_EcoRI_rev | GAATTC AACTTG GTGGTG TCTGGG AC |
| pGEX4T1_AT5G58150kin_BamH1_for | TACGGATCCATCAAGCAAGCAACGCAAATCCCTG |
| pGEX4T1_AT5G58150kin_Sma1_rev | AGTACCCGGGCTAGTAGTTGGGAGAAATGTCTTTG |
| GATEWAY_AT5G58150kin_for | GGGGACAAGTTTGTACAAAAAAGCAGGCTTCATCAAGCAAGCAACGCAAATCCCTG |
| GATEWAY_AT5G58150_for | GGGGACAAGTTGTTACAAAAAAGCAGGCTTCATGAGGTTGTCTCTATGGGGATC |
| GATEWAY_AT5G58150_rev | GGGGACCACTTTGTACAAGAAAGCTGGGTCGTAGTTGGGAGAAATGTCTTTGAG |
| RealTime_At5g58150_for | AGCCAGGAGCCACGCTTAGC |
| RealTime_At5g58150_rev | CGCTCCTGTTCCGGTGGTGTG |
| SALK_093781_rev | GAAAGTTGGATGGGAAAGCTC |
| SALK_093781_for | TATCTCGAGCAAACCATCTGG |
| Ath_Actin_For | GGAATCCACGAGACAACCTATAAC |
| Ath_Actin_Rev | GAAACATTTTCTGTGAACGATTCCT |
| TRM1EcoRIF | TACTGAATTCATGGGGTGTACATCTTCCAAG |
| TRM1SalIR | TACTGTCGACTTAATTAACAAAACACACGGGAC |
| Lba1 | TGGTTCACGTAGTGGGCCATCG |

Table S9. Genes co-expressed with *AT5g58150* based on published data from transcriptomic analysis performed with the Arabidopsis Co-expression Tool (<https://www.michalopoulos.net/act>). Genes of the brassinosteroid pathway, involved in root development as well as cell wall and junction formation, are marked in yellow.

| Rank | *p-*value adjusted for FDR | Hits | Over-representation | Accession | Biologicaprocess |
| --- | --- | --- | --- | --- | --- |
| 1 | 4.9⋅10^-14^ | 122/407 (30.0%) | 2.1 | [GO:0019748](http://amigo.geneontology.org/amigo/term/GO:0019748) | secondary metabolic process |
| 2 | 8.6⋅10^-14^ | 162/613 (26.4%) | 1.9 | [GO:0071554](http://amigo.geneontology.org/amigo/term/GO:0071554) | cell wall organization or biogenesis |
| 3 | 2.7⋅10^-13^ | 77/214 (36.0%) | 2.6 | [GO:0009636](http://amigo.geneontology.org/amigo/term/GO:0009636) | response to toxic substance |
| 4 | 6.2⋅10^-11^ | 35/67 (52.2%) | 3.7 | [GO:0042744](http://amigo.geneontology.org/amigo/term/GO:0042744) | hydrogen peroxide catabolic process |
| 5 | 9.8⋅10^-11^ | 58/155 (37.4%) | 2.7 | [GO:0009698](http://amigo.geneontology.org/amigo/term/GO:0009698) | phenylpropanoid metabolic process |
| 6 | 8.5⋅10^-10^ | 70/216 (32.4%) | 2.3 | [GO:0071669](http://amigo.geneontology.org/amigo/term/GO:0071669) | plant-type cell wall organization or biogenesis |
| 7 | 8.5⋅10^-10^ | 65/193 (33.7%) | 2.4 | [GO:0042546](http://amigo.geneontology.org/amigo/term/GO:0042546) | cell wall biogenesis |
| 8 | 9.3⋅10^-10^ | 38/84 (45.2%) | 3.2 | [GO:0042743](http://amigo.geneontology.org/amigo/term/GO:0042743) | hydrogen peroxide metabolic process |
| 9 | 3.8⋅10^-9^ | 30/59 (50.8%) | 3.6 | [GO:0009834](http://amigo.geneontology.org/amigo/term/GO:0009834) | plant-type secondary cell wall biogenesis |
| 10 | 4.0⋅10^-9^ | 52/144 (36.1%) | 2.6 | [GO:0098754](http://amigo.geneontology.org/amigo/term/GO:0098754) | detoxification |
| 11 | 1.0⋅10^-8^ | 312/1597 (19.5%) | 1.4 | [GO:0055114](http://amigo.geneontology.org/amigo/term/GO:0055114) | oxidation-reduction process |
| 12 | 5.9⋅10^-8^ | 48/137 (35.0%) | 2.5 | [GO:0098869](http://amigo.geneontology.org/amigo/term/GO:0098869) | cellular oxidant detoxification |
| 13 | 5.9⋅10^-8^ | 48/137 (35.0%) | 2.5 | [GO:1990748](http://amigo.geneontology.org/amigo/term/GO:1990748) | cellular detoxification |
| 14 | 1.0⋅10^-7^ | 44/122 (36.1%) | 2.6 | [GO:0009699](http://amigo.geneontology.org/amigo/term/GO:0009699) | phenylpropanoid biosynthetic process |
| 15 | 1.8⋅10^-7^ | 101/402 (25.1%) | 1.8 | [GO:0048364](http://amigo.geneontology.org/amigo/term/GO:0048364) | root development |
| 16 | 2.1⋅10^-7^ | 101/404 (25.0%) | 1.8 | [GO:0022622](http://amigo.geneontology.org/amigo/term/GO:0022622) | root system development |
| 17 | 2.1⋅10^-7^ | 77/280 (27.5%) | 2.0 | [GO:0044550](http://amigo.geneontology.org/amigo/term/GO:0044550) | secondary metabolite biosynthetic process |
| 18 | 2.6⋅10^-7^ | 206/1002 (20.6%) | 1.5 | [GO:0006468](http://amigo.geneontology.org/amigo/term/GO:0006468) | protein phosphorylation |
| 19 | 2.2⋅10^-6^ | 115/499 (23.0%) | 1.6 | [GO:0045229](http://amigo.geneontology.org/amigo/term/GO:0045229) | external encapsulating structure organization |
| 20 | 2.4⋅10^-6^ | 109/467 (23.3%) | 1.7 | [GO:0071555](http://amigo.geneontology.org/amigo/term/GO:0071555) | cell wall organization |
| 21 | 3.9⋅10^-6^ | 44/137 (32.1%) | 2.3 | [GO:0044036](http://amigo.geneontology.org/amigo/term/GO:0044036) | cell wall macromolecule metabolic process |
| 22 | 9.5⋅10^-6^ | 33/92 (35.9%) | 2.6 | [GO:0010054](http://amigo.geneontology.org/amigo/term/GO:0010054) | trichoblast differentiation |
| 23 | 1.9⋅10^-5^ | 22/50 (44.0%) | 3.1 | [GO:0071241](http://amigo.geneontology.org/amigo/term/GO:0071241) | cellular response to inorganic substance |
| 24 | 2.4⋅10^-5^ | 31/87 (35.6%) | 2.5 | [GO:0010410](http://amigo.geneontology.org/amigo/term/GO:0010410) | hemicellulose metabolic process |
| 25 | 2.4⋅10^-5^ | 43/141 (30.5%) | 2.2 | [GO:0072593](http://amigo.geneontology.org/amigo/term/GO:0072593) | reactive oxygen species metabolic process |
| 26 | 2.9⋅10^-5^ | 822/5155 (15.9%) | 1.1 | [GO:0050896](http://amigo.geneontology.org/amigo/term/GO:0050896) | response to stimulus |
| 27 | 3.3⋅10^-5^ | 40/129 (31.0%) | 2.2 | [GO:0009832](http://amigo.geneontology.org/amigo/term/GO:0009832) | plant-type cell wall biogenesis |
| 28 | 3.3⋅10^-5^ | 14/24 (58.3%) | 4.2 | [GO:1902170](http://amigo.geneontology.org/amigo/term/GO:1902170) | cellular response to reactive nitrogen species |
| 29 | 3.8⋅10^-5^ | 435/2538 (17.1%) | 1.2 | [GO:0042221](http://amigo.geneontology.org/amigo/term/GO:0042221) | response to chemical |
| 30 | 4.8⋅10^-5^ | 30/86 (34.9%) | 2.5 | [GO:0048469](http://amigo.geneontology.org/amigo/term/GO:0048469) | cell maturation |
| 31 | 4.8⋅10^-5^ | 30/86 (34.9%) | 2.5 | [GO:0048764](http://amigo.geneontology.org/amigo/term/GO:0048764) | trichoblast maturation |
| 32 | 4.8⋅10^-5^ | 30/86 (34.9%) | 2.5 | [GO:0048765](http://amigo.geneontology.org/amigo/term/GO:0048765) | root hair cell differentiation |
| 33 | 6.5⋅10^-5^ | 34/105 (32.4%) | 2.3 | [GO:0010053](http://amigo.geneontology.org/amigo/term/GO:0010053) | root epidermal cell differentiation |
| 34 | 8.8⋅10^-5^ | 29/84 (34.5%) | 2.5 | [GO:0009808](http://amigo.geneontology.org/amigo/term/GO:0009808) | lignin metabolic process |
| 35 | 8.9⋅10^-5^ | 55/209 (26.3%) | 1.9 | [GO:0010015](http://amigo.geneontology.org/amigo/term/GO:0010015) | root morphogenesis |
| 36 | 1.1⋅10^-4^ | 12/20 (60.0%) | 4.3 | [GO:0071732](http://amigo.geneontology.org/amigo/term/GO:0071732) | cellular response to nitric oxide |
| 37 | 1.3⋅10^-4^ | 17/37 (45.9%) | 3.3 | [GO:0045491](http://amigo.geneontology.org/amigo/term/GO:0045491) | xylan metabolic process |
| 38 | 1.5⋅10^-4^ | 18/41 (43.9%) | 3.1 | [GO:0009407](http://amigo.geneontology.org/amigo/term/GO:0009407) | toxin catabolic process |
| 39 | 1.5⋅10^-4^ | 18/41 (43.9%) | 3.1 | [GO:0090487](http://amigo.geneontology.org/amigo/term/GO:0090487) | secondary metabolite catabolic process |
| 40 | 1.6⋅10^-4^ | 20/49 (40.8%) | 2.9 | [GO:0048767](http://amigo.geneontology.org/amigo/term/GO:0048767) | root hair elongation |
| 41 | 1.7⋅10^-4^ | 10/15 (66.7%) | 4.8 | [GO:0046271](http://amigo.geneontology.org/amigo/term/GO:0046271) | phenylpropanoid catabolic process |
| 42 | 1.7⋅10^-4^ | 10/15 (66.7%) | 4.8 | [GO:0046274](http://amigo.geneontology.org/amigo/term/GO:0046274) | lignin catabolic process |
| 43 | 1.7⋅10^-4^ | 10/15 (66.7%) | 4.8 | [GO:0009806](http://amigo.geneontology.org/amigo/term/GO:0009806) | lignan metabolic process |
| 44 | 1.7⋅10^-4^ | 10/15 (66.7%) | 4.8 | [GO:0009807](http://amigo.geneontology.org/amigo/term/GO:0009807) | lignan biosynthetic process |
| 45 | 1.8⋅10^-4^ | 12/21 (57.1%) | 4.1 | [GO:0071731](http://amigo.geneontology.org/amigo/term/GO:0071731) | response to nitric oxide |
| 46 | 1.9⋅10^-4^ | 11/18 (61.1%) | 4.4 | [GO:0010345](http://amigo.geneontology.org/amigo/term/GO:0010345) | suberin biosynthetic process |
| 47 | 2.0⋅10^-4^ | 256/1420 (18.0%) | 1.3 | [GO:0016310](http://amigo.geneontology.org/amigo/term/GO:0016310) | phosphorylation |
| 48 | 2.3⋅10^-4^ | 14/28 (50.0%) | 3.6 | [GO:0045492](http://amigo.geneontology.org/amigo/term/GO:0045492) | xylan biosynthetic process |
| 49 | 2.7⋅10^-4^ | 6/6 (100.0%) | 7.1 | [GO:0034329](http://amigo.geneontology.org/amigo/term/GO:0034329) | cell junction assembly |
| 50 | 3.0⋅10^-4^ | 24/68 (35.3%) | 2.5 | [GO:0080147](http://amigo.geneontology.org/amigo/term/GO:0080147) | root hair cell development |
| 51 | 3.4⋅10^-4^ | 35/119 (29.4%) | 2.1 | [GO:0090627](http://amigo.geneontology.org/amigo/term/GO:0090627) | plant epidermal cell differentiation |
| 52 | 3.5⋅10^-4^ | 17/40 (42.5%) | 3.0 | [GO:0055088](http://amigo.geneontology.org/amigo/term/GO:0055088) | lipid homeostasis |
| 53 | 4.3⋅10^-4^ | 32/106 (30.2%) | 2.2 | [GO:0010383](http://amigo.geneontology.org/amigo/term/GO:0010383) | cell wall polysaccharide metabolic process |
| 54 | 5.2⋅10^-4^ | 48/187 (25.7%) | 1.8 | [GO:0071369](http://amigo.geneontology.org/amigo/term/GO:0071369) | cellular response to ethylene stimulus |
| 55 | 6.6⋅10^-4^ | 20/54 (37.0%) | 2.6 | [GO:0006749](http://amigo.geneontology.org/amigo/term/GO:0006749) | glutathione metabolic process |
| 56 | 8.1⋅10^-4^ | 34/119 (28.6%) | 2.0 | [GO:0009664](http://amigo.geneontology.org/amigo/term/GO:0009664) | plant-type cell wall organization |
| 57 | 9.1⋅10^-4^ | 15/35 (42.9%) | 3.1 | [GO:0016128](http://amigo.geneontology.org/amigo/term/GO:0016128) | phytosteroid metabolic process |
| 58 | 9.1⋅10^-4^ | 15/35 (42.9%) | 3.1 | [GO:0016131](http://amigo.geneontology.org/amigo/term/GO:0016131) | brassinosteroid metabolic process |
| 59 | 9.6⋅10^-4^ | 17/43 (39.5%) | 2.8 | [GO:0071248](http://amigo.geneontology.org/amigo/term/GO:0071248) | cellular response to metal ion |
| 60 | 9.8⋅10^-4^ | 21/60 (35.0%) | 2.5 | [GO:0019585](http://amigo.geneontology.org/amigo/term/GO:0019585) | glucuronate metabolic process |
| 61 | 9.8⋅10^-4^ | 21/60 (35.0%) | 2.5 | [GO:0052695](http://amigo.geneontology.org/amigo/term/GO:0052695) | cellular glucuronidation |
| 62 | 9.8⋅10^-4^ | 21/60 (35.0%) | 2.5 | [GO:0052696](http://amigo.geneontology.org/amigo/term/GO:0052696) | flavonoid glucuronidation |
| 63 | 9.8⋅10^-4^ | 47/187 (25.1%) | 1.8 | [GO:0009611](http://amigo.geneontology.org/amigo/term/GO:0009611) | response to wounding |
| 64 | 9.8⋅10^-4^ | 11/21 (52.4%) | 3.7 | [GO:2000652](http://amigo.geneontology.org/amigo/term/GO:2000652) | regulation of secondary cell wall biogenesis |
| 65 | 1.2⋅10^-3^ | 6/7 (85.7%) | 6.1 | [GO:0034330](http://amigo.geneontology.org/amigo/term/GO:0034330) | cell junction organization |
| 66 | 1.2⋅10^-3^ | 6/7 (85.7%) | 6.1 | [GO:0045216](http://amigo.geneontology.org/amigo/term/GO:0045216) | cell-cell junction organization |
| 67 | 1.3⋅10^-3^ | 84/396 (21.2%) | 1.5 | [GO:0005976](http://amigo.geneontology.org/amigo/term/GO:0005976) | polysaccharide metabolic process |
| 68 | 1.4⋅10^-3^ | 5/5 (100.0%) | 7.1 | [GO:0007043](http://amigo.geneontology.org/amigo/term/GO:0007043) | cell-cell junction assembly |
| 69 | 1.4⋅10^-3^ | 19/53 (35.8%) | 2.6 | [GO:0009404](http://amigo.geneontology.org/amigo/term/GO:0009404) | toxin metabolic process |
| 70 | 1.5⋅10^-3^ | 208/1159 (17.9%) | 1.3 | [GO:0006952](http://amigo.geneontology.org/amigo/term/GO:0006952) | defense response |
| 71 | 1.5⋅10^-3^ | 48/196 (24.5%) | 1.7 | [GO:0034637](http://amigo.geneontology.org/amigo/term/GO:0034637) | cellular carbohydrate biosynthetic process |
| 72 | 1.6⋅10^-3^ | 63/279 (22.6%) | 1.6 | [GO:0009723](http://amigo.geneontology.org/amigo/term/GO:0009723) | response to ethylene |
| 73 | 1.9⋅10^-3^ | 21/63 (33.3%) | 2.4 | [GO:0006063](http://amigo.geneontology.org/amigo/term/GO:0006063) | uronic acid metabolic process |
| 74 | 2.7⋅10^-3^ | 22/69 (31.9%) | 2.3 | [GO:0036293](http://amigo.geneontology.org/amigo/term/GO:0036293) | response to decreased oxygen levels |
| 75 | 2.9⋅10^-3^ | 12/27 (44.4%) | 3.2 | [GO:0010268](http://amigo.geneontology.org/amigo/term/GO:0010268) | brassinosteroid homeostasis |
| 76 | 3.0⋅10^-3^ | 13/31 (41.9%) | 3.0 | [GO:0016129](http://amigo.geneontology.org/amigo/term/GO:0016129) | phytosteroid biosynthetic process |
| 77 | 3.0⋅10^-3^ | 13/31 (41.9%) | 3.0 | [GO:0016132](http://amigo.geneontology.org/amigo/term/GO:0016132) | brassinosteroid biosynthetic process |
| 78 | 3.1⋅10^-3^ | 14/35 (40.0%) | 2.9 | [GO:0071281](http://amigo.geneontology.org/amigo/term/GO:0071281) | cellular response to iron ion |
| 79 | 3.2⋅10^-3^ | 22/70 (31.4%) | 2.2 | [GO:0070482](http://amigo.geneontology.org/amigo/term/GO:0070482) | response to oxygen levels |
| 80 | 3.5⋅10^-3^ | 9/17 (52.9%) | 3.8 | [GO:0006026](http://amigo.geneontology.org/amigo/term/GO:0006026) | aminoglycan catabolic process |
| 81 | 3.5⋅10^-3^ | 9/17 (52.9%) | 3.8 | [GO:0006030](http://amigo.geneontology.org/amigo/term/GO:0006030) | chitin metabolic process |
| 82 | 3.5⋅10^-3^ | 9/17 (52.9%) | 3.8 | [GO:0006032](http://amigo.geneontology.org/amigo/term/GO:0006032) | chitin catabolic process |
| 83 | 3.5⋅10^-3^ | 9/17 (52.9%) | 3.8 | [GO:0046348](http://amigo.geneontology.org/amigo/term/GO:0046348) | amino sugar catabolic process |
| 84 | 3.5⋅10^-3^ | 9/17 (52.9%) | 3.8 | [GO:1901072](http://amigo.geneontology.org/amigo/term/GO:1901072) | glucosamine-containing compound catabolic process |
| 85 | 3.8⋅10^-3^ | 12/28 (42.9%) | 3.1 | [GO:0036294](http://amigo.geneontology.org/amigo/term/GO:0036294) | cellular response to decreased oxygen levels |
| 86 | 3.8⋅10^-3^ | 12/28 (42.9%) | 3.1 | [GO:0071453](http://amigo.geneontology.org/amigo/term/GO:0071453) | cellular response to oxygen levels |
| 87 | 5.3⋅10^-3^ | 11/25 (44.0%) | 3.1 | [GO:0071456](http://amigo.geneontology.org/amigo/term/GO:0071456) | cellular response to hypoxia |
| 88 | 5.6⋅10^-3^ | 5/6 (83.3%) | 5.9 | [GO:0006868](http://amigo.geneontology.org/amigo/term/GO:0006868) | glutamine transport |
| 89 | 5.6⋅10^-3^ | 183/1030 (17.8%) | 1.3 | [GO:0005975](http://amigo.geneontology.org/amigo/term/GO:0005975) | carbohydrate metabolic process |
| 90 | 6.0⋅10^-3^ | 53/237 (22.4%) | 1.6 | [GO:0006357](http://amigo.geneontology.org/amigo/term/GO:0006357) | regulation of transcription by RNA polymerase II |
| 91 | 6.2⋅10^-3^ | 27/98 (27.6%) | 2.0 | [GO:1990267](http://amigo.geneontology.org/amigo/term/GO:1990267) | response to transition metal nanoparticle |
| 92 | 7.2⋅10^-3^ | 4/4 (100.0%) | 7.1 | [GO:0010683](http://amigo.geneontology.org/amigo/term/GO:0010683) | tricyclic triterpenoid metabolic process |
| 93 | 7.8⋅10^-3^ | 7/12 (58.3%) | 4.2 | [GO:0032973](http://amigo.geneontology.org/amigo/term/GO:0032973) | amino acid export across plasma membrane |
| 94 | 7.8⋅10^-3^ | 7/12 (58.3%) | 4.2 | [GO:0010413](http://amigo.geneontology.org/amigo/term/GO:0010413) | glucuronoxylan metabolic process |
| 95 | 7.8⋅10^-3^ | 7/12 (58.3%) | 4.2 | [GO:0010417](http://amigo.geneontology.org/amigo/term/GO:0010417) | glucuronoxylan biosynthetic process |
| 96 | 7.8⋅10^-3^ | 6/9 (66.7%) | 4.8 | [GO:0033609](http://amigo.geneontology.org/amigo/term/GO:0033609) | oxalate metabolic process |
| 97 | 7.8⋅10^-3^ | 16/47 (34.0%) | 2.4 | [GO:0015893](http://amigo.geneontology.org/amigo/term/GO:0015893) | drug transport |
| 98 | 8.2⋅10^-3^ | 33/131 (25.2%) | 1.8 | [GO:0042445](http://amigo.geneontology.org/amigo/term/GO:0042445) | hormone metabolic process |
| 99 | 8.7⋅10^-3^ | 9/19 (47.4%) | 3.4 | [GO:1901071](http://amigo.geneontology.org/amigo/term/GO:1901071) | glucosamine-containing compound metabolic process |
| 100 | 9.6⋅10^-3^ | 16/48 (33.3%) | 2.4 | [GO:0042493](http://amigo.geneontology.org/amigo/term/GO:0042493) | response to drug |
| 101 | 1.0⋅10^-2^ | 19/62 (30.6%) | 2.2 | [GO:0001666](http://amigo.geneontology.org/amigo/term/GO:0001666) | response to hypoxia |
| 102 | 1.1⋅10^-2^ | 162/912 (17.8%) | 1.3 | [GO:0009607](http://amigo.geneontology.org/amigo/term/GO:0009607) | response to biotic stimulus |
| 103 | 1.1⋅10^-2^ | 102/536 (19.0%) | 1.4 | [GO:0035556](http://amigo.geneontology.org/amigo/term/GO:0035556) | intracellular signal transduction |
| 104 | 1.4⋅10^-2^ | 15/45 (33.3%) | 2.4 | [GO:0070592](http://amigo.geneontology.org/amigo/term/GO:0070592) | cell wall polysaccharide biosynthetic process |
| 105 | 1.4⋅10^-2^ | 82/418 (19.6%) | 1.4 | [GO:0006979](http://amigo.geneontology.org/amigo/term/GO:0006979) | response to oxidative stress |
| 106 | 1.4⋅10^-2^ | 53/247 (21.5%) | 1.5 | [GO:0044264](http://amigo.geneontology.org/amigo/term/GO:0044264) | cellular polysaccharide metabolic process |
| 107 | 1.6⋅10^-2^ | 61/295 (20.7%) | 1.5 | [GO:0009620](http://amigo.geneontology.org/amigo/term/GO:0009620) | response to fungus |
| 108 | 1.7⋅10^-2^ | 15/46 (32.6%) | 2.3 | [GO:0044038](http://amigo.geneontology.org/amigo/term/GO:0044038) | cell wall macromolecule biosynthetic process |
| 109 | 1.7⋅10^-2^ | 15/46 (32.6%) | 2.3 | [GO:0070589](http://amigo.geneontology.org/amigo/term/GO:0070589) | cellular component macromolecule biosynthetic process |
| 110 | 1.9⋅10^-2^ | 201/1177 (17.1%) | 1.2 | [GO:0009605](http://amigo.geneontology.org/amigo/term/GO:0009605) | response to external stimulus |
| 111 | 1.9⋅10^-2^ | 14/42 (33.3%) | 2.4 | [GO:0006855](http://amigo.geneontology.org/amigo/term/GO:0006855) | drug transmembrane transport |
| 112 | 2.2⋅10^-2^ | 35/150 (23.3%) | 1.7 | [GO:0033692](http://amigo.geneontology.org/amigo/term/GO:0033692) | cellular polysaccharide biosynthetic process |
| 113 | 2.5⋅10^-2^ | 4/5 (80.0%) | 5.7 | [GO:0033358](http://amigo.geneontology.org/amigo/term/GO:0033358) | UDP-L-arabinose biosynthetic process |
| 114 | 2.5⋅10^-2^ | 4/5 (80.0%) | 5.7 | [GO:0045226](http://amigo.geneontology.org/amigo/term/GO:0045226) | extracellular polysaccharide biosynthetic process |
| 115 | 2.5⋅10^-2^ | 4/5 (80.0%) | 5.7 | [GO:0045227](http://amigo.geneontology.org/amigo/term/GO:0045227) | capsule polysaccharide biosynthetic process |
| 116 | 2.5⋅10^-2^ | 4/5 (80.0%) | 5.7 | [GO:0045230](http://amigo.geneontology.org/amigo/term/GO:0045230) | capsule organization |
| 117 | 2.5⋅10^-2^ | 4/5 (80.0%) | 5.7 | [GO:0046379](http://amigo.geneontology.org/amigo/term/GO:0046379) | extracellular polysaccharide metabolic process |
| 118 | 2.5⋅10^-2^ | 11/30 (36.7%) | 2.6 | [GO:0016998](http://amigo.geneontology.org/amigo/term/GO:0016998) | cell wall macromolecule catabolic process |
| 119 | 2.5⋅10^-2^ | 11/30 (36.7%) | 2.6 | [GO:1903338](http://amigo.geneontology.org/amigo/term/GO:1903338) | regulation of cell wall organization or biogenesis |
| 120 | 2.5⋅10^-2^ | 446/2826 (15.8%) | 1.1 | [GO:0006950](http://amigo.geneontology.org/amigo/term/GO:0006950) | response to stress |
| 121 | 2.5⋅10^-2^ | 8/18 (44.4%) | 3.2 | [GO:0005986](http://amigo.geneontology.org/amigo/term/GO:0005986) | sucrose biosynthetic process |
| 122 | 2.7⋅10^-2^ | 34/147 (23.1%) | 1.6 | [GO:0042545](http://amigo.geneontology.org/amigo/term/GO:0042545) | cell wall modification |
| 123 | 2.9⋅10^-2^ | 25/99 (25.3%) | 1.8 | [GO:0042446](http://amigo.geneontology.org/amigo/term/GO:0042446) | hormone biosynthetic process |
| 124 | 3.0⋅10^-2^ | 15/49 (30.6%) | 2.2 | [GO:0034614](http://amigo.geneontology.org/amigo/term/GO:0034614) | cellular response to reactive oxygen species |
| 125 | 3.0⋅10^-2^ | 119/662 (18.0%) | 1.3 | [GO:0030154](http://amigo.geneontology.org/amigo/term/GO:0030154) | cell differentiation |
| 126 | 3.4⋅10^-2^ | 299/1845 (16.2%) | 1.2 | [GO:0007154](http://amigo.geneontology.org/amigo/term/GO:0007154) | cell communication |
| 127 | 3.6⋅10^-2^ | 316/1962 (16.1%) | 1.1 | [GO:0006796](http://amigo.geneontology.org/amigo/term/GO:0006796) | phosphate-containing compound metabolic process |
| 128 | 3.6⋅10^-2^ | 3/3 (100.0%) | 7.1 | [GO:0080165](http://amigo.geneontology.org/amigo/term/GO:0080165) | callose deposition in phloem sieve plate |
| 129 | 3.6⋅10^-2^ | 3/3 (100.0%) | 7.1 | [GO:0010045](http://amigo.geneontology.org/amigo/term/GO:0010045) | response to nickel cation |
| 130 | 3.6⋅10^-2^ | 3/3 (100.0%) | 7.1 | [GO:0014074](http://amigo.geneontology.org/amigo/term/GO:0014074) | response to purine-containing compound |
| 131 | 3.6⋅10^-2^ | 3/3 (100.0%) | 7.1 | [GO:0080003](http://amigo.geneontology.org/amigo/term/GO:0080003) | thalianol metabolic process |
| 132 | 4.0⋅10^-2^ | 133/758 (17.5%) | 1.3 | [GO:0071495](http://amigo.geneontology.org/amigo/term/GO:0071495) | cellular response to endogenous stimulus |
| 133 | 4.0⋅10^-2^ | 44/208 (21.2%) | 1.5 | [GO:0090558](http://amigo.geneontology.org/amigo/term/GO:0090558) | plant epidermis development |
| 134 | 4.1⋅10^-2^ | 217/1306 (16.6%) | 1.2 | [GO:0009056](http://amigo.geneontology.org/amigo/term/GO:0009056) | catabolic process |
| 135 | 4.1⋅10^-2^ | 14/46 (30.4%) | 2.2 | [GO:0010039](http://amigo.geneontology.org/amigo/term/GO:0010039) | response to iron ion |
| 136 | 4.1⋅10^-2^ | 317/1974 (16.1%) | 1.1 | [GO:0006793](http://amigo.geneontology.org/amigo/term/GO:0006793) | phosphorus metabolic process |
| 137 | 4.2⋅10^-2^ | 29/124 (23.4%) | 1.7 | [GO:0046777](http://amigo.geneontology.org/amigo/term/GO:0046777) | protein autophosphorylation |
| 138 | 4.3⋅10^-2^ | 56/280 (20.0%) | 1.4 | [GO:0030001](http://amigo.geneontology.org/amigo/term/GO:0030001) | metal ion transport |
| 139 | 4.5⋅10^-2^ | 53/263 (20.2%) | 1.4 | [GO:0006366](http://amigo.geneontology.org/amigo/term/GO:0006366) | transcription by RNA polymerase II |
| 140 | 4.5⋅10^-2^ | 138/794 (17.4%) | 1.2 | [GO:0099402](http://amigo.geneontology.org/amigo/term/GO:0099402) | plant organ development |
| 141 | 4.5⋅10^-2^ | 9/24 (37.5%) | 2.7 | [GO:0006022](http://amigo.geneontology.org/amigo/term/GO:0006022) | aminoglycan metabolic process |
| 142 | 4.5⋅10^-2^ | 7/16 (43.8%) | 3.1 | [GO:0034755](http://amigo.geneontology.org/amigo/term/GO:0034755) | iron ion transmembrane transport |
| 143 | 4.5⋅10^-2^ | 40/187 (21.4%) | 1.5 | [GO:0007166](http://amigo.geneontology.org/amigo/term/GO:0007166) | cell surface receptor signaling pathway |
