# Supplementary material for: Phototropin2 3’UTR overlaps with the AT5G58150 gene encoding an inactive RLK kinase
Source: BMC Plant Biol. 2024 Jan 18;24:55. doi: 10.1186/s12870-024-04732-2 (PMC10795372; doi:10.1186/s12870-024-04732-2)
Supplement: Supplementary file 2 — Additional file 2: Table S1. Results of the statistical analysis of the differences between wild type plants and at5g58150/ AT5G58150-GFP lines in fraction of non-germinated seeds two (A, C) or three (B, D) days after end of stratification on media supplemented with different concentrations of NaCl (A, B) or mannitol (C, D). A logistic regression model was fitted using the glm command of the R software. Type III analysis of deviance was performed using the Anova command of the car package, with the effects coding of factor levels. Differences between natural logarithms of odds ratios were tested with the glhtcommand of the multcomp package, with a custom contrast matrix. p values are adjusted for multiple comparisons using Hommel’s method. Table S2. Results of the statistical analysis of the differences between wild type plants and at5g58150/ AT5G58150-GFP lines in fraction of seeds that germinated (radicles emerged), but the seedlings did not open cotyledons. Table S3. Results of the statistical analysis of the differences in mean fraction of seeds that germinated and opened cotyledons, two (A, C) or three (B, D) days after end of stratification on media supplemented with different concentrations of NaCl (A, B) or mannitol (C, D). Table S4. Results of the statistical analysis of the differences in mean root length (A), the number of lateral roots per cm of the primary root (B) and lateral root count (C) of 9-day-old Arabidopsis seedlings. Table S5. Results of the statistical analysis of the interaction contrasts (differences of differences) for lateral root density (the number of lateral roots per cm of the primary root) of 9-day-old Arabidopsis seedlings. [file 12870_2024_4732_MOESM2_ESM.docx]

Table S1. Results of the statistical analysis of the differences between wild type plants and *at5g58150*/ AT5G58150-GFP lines in fraction of non-germinated seeds two (A, C) or three (B, D) days after end of stratification on media supplemented with different concentrations of NaCl (A, B) or mannitol (C, D). A logistic regression model was fitted using the *glm* command of the R software. Type III analysis of deviance was performed using the *Anova* command of the *car* package, with the effects coding of factor levels. Differences between natural logarithms of odds ratios were tested with the *glht* command of the *multcomp* package, with a custom contrast matrix. *p* values are adjusted for multiple comparisons using Hommel’s method.

**A**

| Analysis of Deviance Table (Type III tests) | | | |
| --- | --- | --- | --- |
|  |  |  |  |
| Response: germinationNaClResponse2Days | | | |
|  | χ^2^ | df | Pr(>χ^2^) |
| Medium | 1234.13 | 2 | < 2.2·10^-16^ *** |
| Line | 7.85 | 2 | 0.019769 * |
| Medium:Line | 13.31 | 4 | 0.009838 ** |

| Fit: glm(formula = response ~ Medium * Line, family = binomial) | | | | |
| --- | --- | --- | --- | --- |
|  |  |  |  |  |
| Hypothesis | Estimate | Standard Error | z value | *p­_adjusted_* |
| NaCl 0 mM: *at5g58150*- WT== 0 | 0.74295 | 0.36511 | 2.035 | 0.1256 |
| NaCl 0 mM: AT5G58150-GFP - WT = 0 | 0.48412 | 0.37969 | 1.275 | 0.4046 |
| NaCl 100 mM: *at5g58150*- WT = 0 | 0.75267 | 0.26888 | 2.799 | 0.0280 * |
| NaCl 100 mM: AT5G58150-GFP - WT = 0 | 0.70356 | 0.27067 | 2.599 | 0.0467 * |
| NaCl 200 mM: *at5g58150*- WT = 0 | -0.08897 | 0.23726 | -0.375 | 0.7077 |
| NaCl 200 mM: AT5G58150-GFP - WT = 0 | -0.45139 | 0.22468 | -2.009 | 0.1336 |

**B**

| Analysis of Deviance Table (Type III tests) | | | |
| --- | --- | --- | --- |
|  |  |  |  |
| Response: germinationNaClResponse3Days | | | |
|  | χ^2^ | df | Pr(>χ^2^) |
| Medium | 387.42 | 2 | <2.2·10^-16^ *** |
| Line | 18.07 | 2 | 0.0001189 *** |
| Medium:Line | 18.15 | 4 | 0.001154 ** |

| Fit: glm(formula = response ~ Medium * Line, family = binomial) | | | | |
| --- | --- | --- | --- | --- |
|  |  |  |  |  |
| Hypothesis | Estimate | Standard Error | z value | *p­_adjusted_* |
| NaCl 0 mM: *at5g58150*- WT = 0 | 0.68667 | 0.40098 | 1.712 | 0.239 |
| NaCl 0 mM: AT5G58150-GFP - WT = 0 | 0.6243 | 0.40456 | 1.543 | 0.246 |
| NaCl 100 mM: *at5g58150*- WT = 0 | 0.47181 | 0.28409 | 1.661 | 0.239 |
| NaCl 100 mM: AT5G58150-GFP - WT = 0 | 0.40412 | 0.28705 | 1.408 | 0.318 |
| NaCl 200 mM: *at5g58150*- WT = 0 | 0.08392 | 0.18325 | 0.458 | 0.647 |
| NaCl 200 mM: AT5G58150-GFP - WT = 0 | 1.16902 | 0.18217 | 6.417 | 8.33·10^-10^ *** |

**C**

| Analysis of Deviance Table (Type III tests) | | | |
| --- | --- | --- | --- |
|  |  |  |  |
| Response: germinationMannitolResponse2Days | | | |
|  | χ^2^ | df | Pr(>χ^2^) |
| Medium | 731.79 | 2 | < 2.2·10^-16^ *** |
| Line | 17.63 | 2 | 0.0001483 *** |
| Medium:Line | 15.669 | 4 | 0.003498 ** |

| Fit: glm(formula = response ~ Medium * Line, family = binomial) | | | | |
| --- | --- | --- | --- | --- |
|  |  |  |  |  |
| Hypothesis | Estimate | Standard Error | z value | *p­_adjusted_* |
| Mannitol 0 mM: *at5g58150*- WT = 0 | 0.7687 | 0.3461 | 2.221 | 0.08989 . |
| Mannitol 0 mM: AT5G58150-GFP - WT = 0 | 0.645 | 0.3527 | 1.829 | 0.09891 . |
| Mannitol 200 mM: *at5g58150*- WT = 0 | 0.818 | 0.3326 | 2.459 | 0.06585 . |
| Mannitol 200 mM: AT5G58150-GFP - WT = 0 | 1.0976 | 0.3221 | 3.408 | 0.00393 ** |
| Mannitol 400 mM: *at5g58150*- WT = 0 | -0.247 | 0.1497 | -1.65 | 0.09891 . |
| Mannitol 400 mM: AT5G58150-GFP - WT = 0 | 0.2753 | 0.1486 | 1.852 | 0.09891 . |

**D**

| Analysis of Deviance Table (Type III tests) | | | |
| --- | --- | --- | --- |
|  |  |  |  |
| Response: germinationMannitolResponse3Days | | | |
|  | χ^2^ | df | Pr(>χ^2^) |
| Medium | 105.623 | 2 | < 2.2·10^-16^ *** |
| Line | 10.081 | 2 | 0.006472 ** |
| Medium:Line | 10.1457 | 4 | 0.03804 * |

| Simultaneous Tests for General Linear Hypotheses | | | | |
| --- | --- | --- | --- | --- |
|  |  |  |  |  |
| Fit: glm(formula = response ~ Medium * Line, family = binomial) | | | | |
|  |  |  |  |  |
| Hypothesis | Estimate | Standard Error | z value | *p­_adjusted_* |
| Mannitol 0 mM: *at5g58150*- WT = 0 | 0.817 | 0.3888 | 2.101 | 0.1424 |
| Mannitol 0 mM: AT5G58150-GFP - WT = 0 | 0.8761 | 0.3863 | 2.268 | 0.0934 . |
| Mannitol 200 mM: *at5g58150*- WT = 0 | 0.5822 | 0.3331 | 1.748 | 0.2416 |
| Mannitol 200 mM: AT5G58150-GFP - WT = 0 | 0.77 | 0.3237 | 2.379 | 0.0869 . |
| Mannitol 400 mM: *at5g58150*- WT = 0 | -0.2401 | 0.2006 | -1.197 | 0.4626 |
| Mannitol 400 mM: AT5G58150-GFP - WT = 0 | 0 | 0.1924 | 0 | 1.0000 |

Table S2. Results of the statistical analysis of the differences between wild type plants and *at5g58150*/ AT5G58150-GFP lines in fraction of seeds that germinated (radicles emerged), but the seedlings did not open cotyledons. Germinated seeds were counted two (A, C) or three (B, D) days after end of stratification on media supplemented with different concentrations of NaCl (A, B) or mannitol (C, D). A logistic regression model was fitted using the *glm* command of the R software. Analysis of deviance was performed using the *Anova* command of the *car* package. Differences between natural logarithms of odds ratios were tested with the *glht* command of the *multcomp* package, with a custom contrast matrix. *p* values are adjusted for multiple comparisons with Hommel’s method.

**A**

| Analysis of Deviance Table (Type III tests) | | | |
| --- | --- | --- | --- |
|  |  |  |  |
| Response: dataRootsNaClResponse2Days | | | |
|  | χ^2^ | df | Pr(>χ^2^) |
| Medium | 813.12 | 2 | < 2·10^-16^ *** |
| Line | 0.35 | 2 | 0.84060 |
| Medium:Line | 12.89 | 4 | 0.01185 * |

| Fit: glm(formula = response ~ Medium * Line, family = binomial) | | | | |
| --- | --- | --- | --- | --- |
|  |  |  |  |  |
| Hypothesis | Estimate | Standard Error | z value | *p­_adjusted_* |
| NaCl 0 mM: *at5g58150*- WT = 0 | 0.26075 | 0.22908 | 1.138 | 0.5100 |
| NaCl 0 mM: AT5G58150-GFP - WT = 0 | 0.30334 | 0.22758 | 1.333 | 0.3825 |
| NaCl 100 mM: *at5g58150*- WT = 0 | -0.30294 | 0.22382 | -1.354 | 0.3825 |
| NaCl 100 mM: AT5G58150-GFP - WT = 0 | -0.5429 | 0.21787 | -2.492 | 0.0763 . |
| NaCl 200 mM: *at5g58150*- WT = 0 | 0.08897 | 0.23726 | 0.375 | 0.7077 |
| NaCl 200 mM: AT5G58150-GFP - WT = 0 | 0.45139 | 0.22468 | 2.009 | 0.2227 |

**B**

| Analysis of Deviance Table (Type III tests) | | | |
| --- | --- | --- | --- |
|  |  |  |  |
| Response: dataRootsNaClResponse3Days | | | |
|  | χ^2^ | df | Pr(>χ^2^) |
| Medium | 690.36 | 2 | < 2.2·10^-16^ *** |
| Line | 1.22 | 2 | 0.5427 |
| Medium:Line | 78.853 | 4 | 3.048·10^-16^ *** |

| Fit: glm(formula = response ~ Medium * Line, family = binomial) | | | | |
| --- | --- | --- | --- | --- |
|  |  |  |  |  |
| Hypothesis | Estimate | Standard. Error | z value | *p­_adjusted_* |
| NaCl 0 mM: *at5g58150*- WT = 0 | -0.40158 | 0.91711 | -0.438 | 0.661 |
| NaCl 0 mM: AT5G58150-GFP - WT = 0 | -0.40547 | 0.9171 | -0.442 | 0.661 |
| NaCl 100 mM: *at5g58150*- WT = 0 | 1.26956 | 0.20638 | 6.151 | 3.84·10^-9^ *** |
| NaCl 100 mM: AT5G58150-GFP - WT = 0 | 1.19046 | 0.20688 | 5.754 | 3.48·10^-8^ *** |
| NaCl 200 mM: *at5g58150*- WT = 0 | -0.08392 | 0.18325 | -0.458 | 0.661 |
| NaCl 200 mM: AT5G58150-GFP - WT = 0 | -1.16902 | 0.18217 | -6.417 | 8.33·10^-10^ *** |

**C**

| Analysis of Deviance Table (Type III tests) | | | |
| --- | --- | --- | --- |
|  |  |  |  |
| Response: dataRootsMannitolResponse2Days | | | |
|  | χ^2^ | df | Pr(>χ^2^) |
| Medium | 545.14 | 2 | < 2.2·10^-16^ *** |
| Line | 73.92 | 2 | < 2.2·10^-16^ *** |
| Medium:Line | 63.549 | 4 | 5.200·10^-13^ *** |

| Fit: glm(formula = response ~ Medium * Line, family = binomial) | | | | | |
| --- | --- | --- | --- | --- | --- |
|  | |  |  |  | |
| Hypothesis | Estimate | Standard Error | z value | *p­_adjusted_* |  |
| Mannitol 0 mM: *at5g58150*- WT = 0 | 1.0329 | 0.2123 | 4.865 | 3.44·10^-6^ *** | |
| Mannitol 0 mM: AT5G58150-GFP - WT = 0 | 1.4828 | 0.2061 | 7.194 | 3.79·10^-12^ *** | |
| Mannitol 200 mM: *at5g58150*- WT = 0 | 0.9725 | 0.1632 | 5.959 | 1.02·10^-8^ *** | |
| Mannitol 200 mM: AT5G58150-GFP - WT = 0 | 0.998 | 0.1647 | 6.06 | 6.36·10^-9^ *** | |
| Mannitol 400 mM: *at5g58150*- WT = 0 | 0.247 | 0.1497 | 1.65 | 0.0989 . | |
| Mannitol 400 mM: AT5G58150-GFP - WT = 0 | -0.2753 | 0.1486 | -1.852 | 0.0989 . | |

**D**

| Analysis of Deviance Table (Type III tests) | | | |
| --- | --- | --- | --- |
|  |  |  |  |
| Response: dataRootsMannitolResponse3Days | | | |
|  | χ^2^ | df | Pr(>χ^2^) |
| Medium | 2072.85 | 2 | < 2.2·10^-16^ *** |
| Line | 3.15 | 2 | 0.2074 |
| Medium:Line | 40.73 | 4 | 3.056e-08 *** |

| Fit: glm(formula = response ~ Medium * Line, family = binomial) | | | | |
| --- | --- | --- | --- | --- |
|  | |  |  |  |
| Hypothesis | Estimate | Standard Error | z value | *p­_adjusted_* |
| Mannitol 0 mM:*at5g58150*- WT = 0 | -0.00551 | 0.81985 | -0.007 | 0.995 |
| Mannitol 0 mM:AT5G58150-GFP - WT = 0 | -0.4027 | 0.91588 | -0.44 | 0.995 |
| Mannitol 200 mM:*at5g58150* - WT = 0 | 1.29137 | 0.25745 | 5.016 | 2.64·10^-6^ *** |
| Mannitol 200 mM:AT5G58150-GFP - WT = 0 | 1.7283 | 0.24988 | 6.916 | 2.78·10^-11^ *** |
| Mannitol 400 mM:*at5g58150* - WT = 0 | 0.19731 | 0.19903 | 0.991 | 0.965 |
| Mannitol 400 mM:AT5G58150-GFP - WT = 0 | -0.14162 | 0.18833 | -0.752 | 0.990 |

Table S3. Results of the statistical analysis of the differences in mean fraction of seeds that germinated and opened cotyledons, two (A, C) or three (B, D) days after end of stratification on media supplemented with different concentrations of NaCl (A, B) or mannitol (C, D). A logistic regression model was fitted using the *glm* command of the R software. Analysis of deviance was performed using the *Anova* command of the *car* package. Differences between natural logarithms of odds ratios were tested with the *glht* command of the *multcomp* package, with a custom contrast matrix. *p* values are adjusted for multiple comparisons with Hommel’s method.

**A**

| Analysis of Deviance Table (Type III tests) | | | |
| --- | --- | --- | --- |
|  |  |  |  |
| Response: dataCotyledonsNaClResponse2Days | | | |
|  | χ^2^ | df | Pr(>χ^2^) |
| Medium | 1448.7 | 2 | < 2·10^-16^ *** |
| Line | 0.0 | 2 | 1.00 |
| Medium:Line | 4.2 | 4 | 0.3791 |

| Fit: glm(formula = response ~ Medium * Line, family = binomial) | | | | |
| --- | --- | --- | --- | --- |
|  |  |  |  |  |
| Hypothesis | Estimate | Standard Error | z value | *p­_adjusted_* |
| NaCl 0 mM: *at5g58150* - WT = 0 | -0.4553 | 0.205 | -2.221 | 0.133 |
| NaCl 0 mM: AT5G58150-GFP - WT = 0 | -0.3934 | 0.206 | -1.909 | 0.225 |
| NaCl 100 mM: *at5g58150* - WT = 0 | -0.7298 | 0.3977 | -1.835 | 0.266 |
| NaCl 100 mM: AT5G58150-GFP - WT = 0 | 0.1607 | 0.3192 | 0.504 | 1.000 |
| NaCl 200 mM: *at5g58150* - WT = 0 | 0.003719 | 2699 | 0 | 1.000 |
| NaCl 200 mM: AT5G58150-GFP - WT = 0 | -0.01474 | 2699 | 0 | 1.000 |

**B**

| Analysis of Deviance Table (Type III tests) | | | |
| --- | --- | --- | --- |
|  |  |  |  |
| Response: dataCotyledonsNaClResponse3Days | | | |
|  | χ^2^ | df | Pr(>χ^2^) |
| Medium | 1786.52 | 2 | <2·10^-16^ *** |
| Line | 0.00 | 2 | 1.0000 |
| Medium:Line | 3.76 | 4 | 0.4391 |

| Fit: glm(formula = response ~ Medium * Line, family = binomial) | | | | |
| --- | --- | --- | --- | --- |
|  |  |  |  |  |
| Hypothesis | Estimate | Standard Error | z value | *p­_adjusted_* |
| NaCl 0 mM: *at5g58150* - WT = 0 | -0.5207 | 0.3644 | -1.429 | 0.459 |
| NaCl 0  mM: AT5G58150-GFP - WT = 0 | -0.4636 | 0.3676 | -1.261 | 0.622 |
| NaCl 100  mM: *at5g58150* - WT = 0 | -1.285 | 0.1885 | -6.819 | 5.51·10^-11^ *** |
| NaCl 100  mM: AT5G58150-GFP - WT = 0 | -1.177 | 0.1881 | -6.256 | 1.97·10^-9^ *** |
| NaCl 200  mM: *at5g58150* - WT = 0 | 0 | 1637 | 0 | 1.000 |
| NaCl 200  mM: AT5G58150-GFP - WT = 0 | -0.01846 | 1637 | 0 | 1.000 |

**C**

| Analysis of Deviance Table (Type III tests) | | | |
| --- | --- | --- | --- |
|  |  |  |  |
| Response: dataCotyledonsMannitolResponse2Days | | | |
|  | χ^2^ | df | Pr(>χ^2^) |
| Medium | 1627.6 | 2 | < 2.2·10^-16^ *** |
| Line | 0.0 | 2 | 1.000 |
| Medium:Line | 1.3 | 4 | 0.862 |

| Fit: glm(formula = response ~ Medium * Line, family = binomial) | | | | |
| --- | --- | --- | --- | --- |
|  |  |  |  |  |
| Hypothesis | Estimate | Standard Error | z value | *p­_adjusted_* |
| Mannitol 0  mM: *at5g58150* - WT = 0 | -1.062 | 0.1895 | -5.603 | 6.33·10^-8^ *** |
| Mannitol 0  mM: AT5G58150-GFP - WT = 0 | -1.426 | 0.1862 | -7.66 | 9.33·10^-14^ *** |
| Mannitol 200  mM: *at5g58150* - WT == 0 | -1.36 | 0.1821 | -7.467 | 3.28·10^-13^ *** |
| Mannitol 200  mM: AT5G58150-GFP - WT = 0 | -1.604 | 0.1945 | -8.244 | 1.33·10^-15^ *** |
| Mannitol 400  mM: *at5g58150* - WT = 0 | 0.005459 | 2281 | 0 | 1.000 |
| Mannitol 400  mM: AT5G58150-GFP - WT = 0 | 0 | 2281 | 0 | 1.000 |

**D**

| Analysis of Deviance Table (Type III tests) | | | |
| --- | --- | --- | --- |
|  |  |  |  |
| Response: dataCotyledonsMannitolResponse3Days | | | |
|  | χ^2^ | df | Pr(>χ^2^) |
| Medium | 2719.44 | 2 | < 2.2·10^-16^ *** |
| Line | 2.60 | 2 | 0.2732 |
| Medium:Line | 29.03 | 4 | 7.723·10^-6^ *** |

| Fit: glm(formula = response ~ Medium * Line, family = binomial) | | | | |
| --- | --- | --- | --- | --- |
|  |  |  |  |  |
| Hypothesis | Estimate | Standard Error | z value | *p­_adjusted_* |
| Mannitol 0  mM: *at5g58150* - WT = 0 | -0.6827 | 0.3503 | -1.949 | 0.154 |
| Mannitol 0  mM: AT5G58150-GFP - WT = 0 | -0.6945 | 0.3503 | -1.983 | 0.142 |
| Mannitol 200  mM: *at5g58150* - WT = 0 | -1.1389 | 0.2106 | -5.408 | 3.19·10^-7^ *** |
| Mannitol 200  mM: AT5G58150-GFP - WT = 0 | -1.5642 | 0.2053 | -7.619 | 1.53·10^-13^ *** |
| Mannitol 400  mM: *at5g58150* - WT = 0 | 15.4705 | 978.3881 | 0.016 | 0.987 |
| Mannitol 400: AT5G58150-GFP - WT = 0 | 16.8735 | 978.3879 | 0.017 | 0.987 |

Table S4. Results of the statistical analysis of the differences in mean root length (A), the number of lateral roots per cm of the primary root (B) and lateral root count (C) of 9-day-old Arabidopsis seedlings. A mixed linear model was fitted using the *lmer* command (A, B) or the *glmer* (C) of the *lme4* package of the R software. The number of lateral roots was assumed to follow Poisson’s distribution. Type III analysis of deviance was performed using the *Anova* command of the *car* package, with the effects coding of factor levels. Differences between group means were tested with the *glht* command of the *multcomp* package, with a custom contrast matrix. *p* values are adjusted for multiple comparisons, using Hommel’s method.

**A**

| Analysis of Deviance Table (Type III Wald χ^2^tests) | | | |
| --- | --- | --- | --- |
|  |  |  |  |
| Response: RootLength | | |  |
|  | χ^2^ | df | Pr(>χ^2^) |
| (Intercept) | 176.387 | 1 | < 2.2·10^-16^ *** |
| Medium | 2232.212 | 2 | < 2.2·10^-16^ *** |
| Line | 22.407 | 2 | 1.362 ·10^-5^ *** |
| Medium:Line | 21.465 | 4 | 0.000256 *** |

| Fit: lmer(formula = RootLength ~ Medium * Line + (1 \| Series), data = dataRoots) | | | | |
| --- | --- | --- | --- | --- |
|  |  |  |  |  |
| Hypothesis | Estimate | Standard Error | z value | *p­_adjusted_* |
| Control: *at5g58150* - WT = 0 | -0.09635 | 0.03827 | -2.517 | 0.0591 . |
| Control:At5G59150-GFP - WT = 0 | 0.17458 | 0.03924 | 4.449 | 5.16 ·10^-5^ *** |
| Mannitol 200 mM: *at5g58150* - WT = 0 | -0.02171 | 0.03768 | -0.576 | 0.7791 |
| Mannitol 200 mM:At5G59150-GFP - WT = 0 | 0.02895 | 0.04057 | 0.713 | 0.7791 |
| NaCl 100 mM: *at5g58150* - WT = 0 | -0.03994 | 0.04055 | -0.985 | 0.7791 |
| NaCl 100 mM:At5G59150-GFP - WT = 0 | -0.01164 | 0.04149 | -0.28 | 0.7791 |

**B**

| Analysis of Deviance Table (Type III Wald χ^2^ tests) | | | |
| --- | --- | --- | --- |
|  |  |  |  |
| Response: RootDensity | | |  |
|  | χ^2^ | df | Pr(>χ^2^) |
| (Intercept) | 368.872 | 1 | < 2.2·10^-16^ *** |
| Medium | 717.459 | 2 | < 2.2·10^-16^ *** |
| Line | 10.585 | 2 | 0.0050301 ** |
| Medium:Line | 18.535 | 4 | 0.0009696 *** |

| Fit: lmer(formula = RootDensity ~ Medium * Line + (1 \| Series), data = dataRoots) | | | | |
| --- | --- | --- | --- | --- |
|  |  |  |  |  |
| Hypothesis | Estimate | Standard Error | z value | *p­_adjusted_* |
| Control: *at5g58150* - WT = 0 | -0.04082 | 0.09635 | -0.424 | 0.67177 |
| Control:At5G59150-GFP - WT = 0 | 0.19957 | 0.09876 | 2.021 | 0.08661 . |
| Mannitol 200 mM: *at5g58150* - WT = 0 | -0.19031 | 0.09486 | -2.006 | 0.08965 . |
| Mannitol 200 mM:At5G59150-GFP - WT = 0 | -0.27673 | 0.10213 | -2.71 | 0.02695 * |
| NaCl 100 mM: *at5g58150* - WT = 0 | -0.26337 | 0.10209 | -2.58 | 0.03956 * |
| NaCl 100 mM:At5G59150-GFP - WT = 0 | -0.36998 | 0.10445 | -3.542 | 0.00238 ** |

**C**

| Analysis of Deviance Table (Type III Wald χ^2^ tests) | | | |
| --- | --- | --- | --- |
|  |  |  |  |
| Response: RootCount | | |  |
|  | χ^2^ | df | Pr(>χ^2^) |
| (Intercept) | 66.272 | 1 | 3.928 ·10^-16^ *** |
| Medium | 789.382 | 2 | < 2.2·10^-16^ *** |
| Line | 11.073 | 2 | 0.0039408 ** |
| Medium:Line | 18.862 | 4 | 0.0008365 *** |

| Fit: glmer(formula = RootCount ~ Medium * Line + (1 \| Series), data = dataRoots, | | | | |
| --- | --- | --- | --- | --- |
| family = poisson(link = "log")) | | |  |  |
|  |  |  |  |  |
| Hypothesis | Estimate | Standard Error | z value | *p­_adjusted_* |
| Control: *at5g58150* - WT = 0 | -0.05482 | 0.06856 | -0.8 | 0.424 |
| Control:At5G59150-GFP - WT = 0 | 0.1788 | 0.06573 | 2.72 | 0.0326 * |
| Mannitol 200 mM: *at5g58150* - WT = 0 | -0.20127 | 0.11209 | -1.796 | 0.1451 |
| Mannitol 200 mM:At5G59150-GFP - WT = 0 | -0.26977 | 0.12386 | -2.178 | 0.0882 . |
| NaCl 100 mM: *at5g58150* - WT = 0 | -0.40289 | 0.17785 | -2.265 | 0.0705 . |
| NaCl 100 mM:At5G59150-GFP - WT = 0 | -0.47907 | 0.1868 | -2.565 | 0.0490 * |

Table S5. Results of the statistical analysis of the interaction contrasts (differences of differences) for lateral root density (the number of lateral roots per cm of the primary root) of 9-day-old Arabidopsis seedlings. Contrasts were tested with the *glht* command of the *multcomp* package, with a custom contrast matrix *K*. *p* values are adjusted for multiple comparisons, using Hommel’s method.

| Fit: lmer(formula = RootDensity ~ Medium * Line + (1 \| Series), data = dataRoots) | | | | | | | | | | | | | |
| --- | --- | --- | --- | --- | --- | --- | --- | --- | --- | --- | --- | --- | --- |
|  | | | | | | | |  | | |  | | |
| Hypothesis | | Estimate | | | Standard Error | | | z value | | | *p­_adjusted_* | | |
| *at5g59150* Control - *at5g59150* Mannitol = WT Control - WT Mannitol | | 0.1495 | | | 0.1352 | | | 1.106 | | | 0.268747 | | |
| At5G59150GFP Control - At5G59150GFP Mannitol = WT Control - WT Mannitol | | 0.4763 | | | 0.1421 | | | 3.353 | | | 0.002401 ** | | |
| *at5g59150* Control - *at5g59150* NaCl = WT Control - WT NaCl | | 0.2225 | | | 0.1404 | | | 1.586 | | | 0.225647 | | |
| At5G59150GFP Control - At5G59150GFP NaCl = WT Control - WT NaCl | | 0.5696 | | | 0.1438 | | | 3.962 | | | 0.000298 *** | | |
| Matrix *K* of interaction contrasts | Group | | | | | | | | | | | | |
|  | Control | | | | | Mannitol 200 mM | | | | NaCl 100 mM | | | |
| Hypothesis | WT | | *at5g59150* | At5G59150-GFP | | WT | *at5g59150* | | At5G59150-GFP | WT | | *at5g59150* | At5G59150-GFP |
| *at5g59150* Control - *at5g59150* Mannitol = WT Control - WT Mannitol | -1 | | 1 | 0 | | 1 | -1 | | 0 | 0 | | 0 | 0 |
| At5G59150GFP Control - At5G59150GFP Mannitol = WT Control - WT Mannitol | -1 | | 0 | 1 | | 1 | 0 | | -1 | 0 | | 0 | 0 |
| *at5g59150* Control - *at5g59150* NaCl = WT Control - WT NaCl | -1 | | 1 | 0 | | 0 | 0 | | 0 | 1 | | -1 | 0 |
| At5G59150GFP Control - At5G59150GFP NaCl = WT Control - WT NaCl | -1 | | 0 | 1 | | 0 | 0 | | 0 | 1 | | 0 | -1 |
